# Supplementary material for: Importance of social inequalities to contact patterns, vaccine uptake, and epidemic dynamics
Source: Nat Commun. 2024 May 16;15:4137. doi: 10.1038/s41467-024-48332-y (PMC11099065; doi:10.1038/s41467-024-48332-y)
Supplement: Supplementary file 1 — Supplementary Information [file 41467_2024_48332_MOESM1_ESM.pdf]

Supplementary Information

Importance of social inequalities to contact patterns, vaccine uptake, and epidemic dynamics

A. Manna, J. Koltai and M. Kársai

Contents

1 Data **2**
1.1 Socio-demographic dimensions 2
1.2 Sample sizes 3
1.3 Data pre-processing 10

2 Robustness checks on the statistical model **10**
2.1 Model on contacts 11
2.1.1 Accounting for the high number of zeros 11
2.1.2 Accounting for the effect of control variables 14
2.2 Model on vaccination 19
2.2.1 Accounting for the effect of NPIs 19

3 Bootstrapping **20**

4 Contacts **20**
4.1 Average number of contacts in the community and in the work layer by sub-groups 20
4.1.1 Relative contact variation 22
4.2 Average number of contacts in the community layer by sub-groups and age groups 23
4.3 Contact Matrices 23
4.3.1 Symmetrization of the decoupled contact matrices 23
4.3.2 Derived contact matrices 24

5 Vaccination uptake **30**

6 Epidemic models **30**
6.1 *Conventional* age-stratified SEIRD 31
6.2 *Extended SEIRD* with vaccination 31

7 Epidemic Simulations **32**
7.1 Impact of different contact patterns 32
7.1.1 Sensitivity analysis of R_0 35
7.2 Impact of different vaccination uptake 39
7.2.1 Sensitivity analysis of vaccination efficacy against infection (g_1) . 40

8 Model Calibration **42**

1 Data

1.1 Socio-demographic dimensions

The MASZK dataset provided us with an extensive set of information on *social-demographic characteristics* of the participants. In this section, we provide a detailed explanation of all the variables used in this work.

- *Education level* which can have four possible levels: low, mid-low, mid-high and high. We considered low-educated individuals with any primary school degrees, mid-low-educated those holding certificates from professional and vocational schools, mid-high-educated those with secondary school, and highly educated with a university education or above (eg. BSc, MSc, PhD).
- *Employment situation* which can be either employed or not-employed. In the not-employed category, we include students and retired individuals.
- *Income level* can have three possible levels: low, mid and high. In particular, individuals were asked to report their *perceived income* with respect to the average using a scale from 1 to 10. We consider low-income individuals who answered from 1 to 4, mid-income those who answered 5 or 6 and high-income those who answered 7 or above. Although this is actually *perceived income*, for conciseness we refer to it in the text as income. Please note that the results concerning this dimension are missing for the 2nd W. This is due to the fact that this variable has been collected only from the 9th data collection onwards.
- *Settlement* refers to the area where individuals live and can be either capital that is Budapest and its districts, rural which includes larger and smaller sizes villages, or urban which includes cities and towns except the capital.
- *Gender* refers to the biological gender and can be either female or male.
- *Chronic disease* is a Boolean dimension indicating if an individual is affected by any chronic disease or not.
- *Acute disease* is a Boolean dimension indicating if an individual is affected by any acute disease or not.
- *Smoking* is a Boolean dimension indicating if an individual is a smoker or not. We consider as *non-smoker* individuals who declared themselves as not-smokers or who stopped, while we consider *smoker*individuals who smoke frequently or occasionally.

1.2 Sample sizes

We report here the sample size by age and the variable of interest. That small variation in the total numbers is due to some missing answers from the participants. In addition, when aggregating by income level we can only show the results starting from the $3rdW$. This is due to the fact that this information has been collected only from the $9th$ data collection. Finally, the overall amounts of individuals in the table by smoking behaviour amount to the adult population.

Education Level

Age Group	Education Level	1st IP	2nd W	3rd W	2nd IP	4th W	5th W
[0 – 5)	low	637	710	376	520	503	604
[5 – 15)	low	1368	1497	849	1086	1113	1314
[15 – 30)	low	483	490	317	422	422	472
	mid-low	122	140	84	121	107	109
	mid-high	547	802	548	514	519	656
	high	358	199	129	90	95	163
[30 – 45)	low	181	74	49	66	43	64
	mid-low	381	261	135	153	147	150
	mid-high	504	508	403	377	358	435
	high	485	670	412	383	471	558
[45 – 60)	low	476	288	200	161	138	235
	mid-low	359	440	250	295	262	405
	mid-high	424	461	331	303	338	416
	high	267	332	258	283	270	281
[60 – 70)	low	413	689	353	297	314	355
	mid-low	302	333	244	196	211	280
	mid-high	316	221	109	126	90	94
	high	34	37	18	48	12	27
[70+)	low	341	269	277	330	346	485
	mid-low	216	189	153	142	184	235
	mid-high	240	148	89	100	114	105
	high	40	27	25	38	5	13
**Total**		8494	8785	5609	6051	6062	7456

Supplementary Table 1: Number of survey participants in the different aggregated periods by age group and the education level

Employment situation

Age Group	Employment situation	1st IP	2nd W	3rd W	2nd IP	4th W	5th W
[0 − 5)	employed	637	710	376	520	503	604
[5 − 15)	employed	1368	1497	849	1086	1113	1314
[15 − 30)	employed	1206	1399	878	947	961	1156
	not employed	304	232	200	200	182	244
[30 − 45)	employed	1127	1241	823	797	863	991
	not employed	424	272	176	182	156	216
[45 − 60)	employed	993	1135	792	808	803	1068
	not employed	533	386	247	234	205	269
[60 − 70)	employed	225	264	176	174	144	178
	not employed	840	1016	548	493	483	578
[70+)	employed	33	30	37	31	28	50
	not employed	804	603	507	579	621	788
**Total**		8494	8785	5609	6051	6062	7456

Supplementary Table 2: Number of survey participants in the different aggregated periods by age group and the education level

Income Level

**Age Group**	**Income Level**	**3rd W**	**2nd IP**	**4th W**	**5th W**
[0 – 5)	high	87	148	173	190
	mid	176	218	219	269
	low	113	154	111	145
[5 – 15)	high	245	275	326	367
	mid	375	515	488	579
	low	229	296	299	368
[15 – 30)	high	291	380	346	432
	mid	562	527	558	674
	low	225	240	239	294
[30 – 45)	high	297	254	308	372
	mid	453	438	462	551
	low	249	287	249	284
[45 – 60)	high	203	224	238	293
	mid	475	498	442	600
	low	361	320	328	444
[60 – 70)	high	82	111	126	152
	mid	302	275	277	325
	low	340	281	224	279
[70+)	high	69	93	132	156
	mid	240	279	273	338
	low	235	238	244	344
**Total**		5609	6051	6062	7456

Supplementary Table 3: Number of survey participants in the different aggregated periods by age group and the income level.

Settlement

Age Group	Settlement	1st IP	2nd W	3rd W	2nd IP	4th W	5th W
[0 – 5)	capital	53	107	57	63	89	109
	rural	226	219	110	194	172	178
	urban	357	384	209	263	242	317
[5 – 15)	capital	137	292	172	144	187	258
	rural	489	474	249	363	359	408
	urban	742	731	428	579	567	647
[15 – 30)	capital	190	172	117	114	116	190
	rural	433	435	302	327	330	381
	urban	887	1023	658	706	697	829
[30 – 45)	capital	178	271	238	149	213	291
	rural	481	406	247	291	261	315
	urban	891	836	513	539	545	600
[45 – 60)	capital	292	359	247	231	192	291
	rural	461	439	285	339	281	372
	urban	773	723	506	472	535	674
[60 – 70)	capital	240	213	85	164	114	83
	rural	377	540	286	224	247	335
	urban	448	527	352	279	266	338
[70+)	capital	241	157	103	135	153	125
	rural	218	175	186	181	243	332
	urban	378	301	253	294	253	381
**Total**		8492	8784	5603	6051	6062	7454

Supplementary Table 4: Number of survey participants in the different aggregated periods by age group and the settlement.

Gender

Age Group	Gender	1st IP	2nd W	3rd W	2nd IP	4th W	5th W
[0 – 5)	Female	282	354	191	257	266	307
	Male	295	356	185	263	237	297
[5 – 15)	Female	626	741	403	561	552	657
	Male	602	756	446	525	561	657
[15 – 30)	Female	683	934	619	705	672	788
	Male	765	697	459	442	471	612
[30 – 45)	Female	720	629	449	429	453	558
	Male	831	884	550	550	566	649
[45 – 60)	Female	882	749	517	458	482	609
	Male	644	772	522	584	526	728
[60 – 70)	Female	600	811	429	367	353	434
	Male	465	469	295	300	274	322
[70+)	Female	490	347	307	388	401	524
	Male	347	286	237	222	248	314
**Total**		8232	8785	5609	6051	6062	7456

Supplementary Table 5: Number of survey participants in the different aggregated periods by age group and gender.

Chronic disease

Age Group	Chronic disease	1st IP	2nd W	3rd W	2nd IP	4th W	5th W
[0 – 5)	no	599	687	369	505	484	574
	yes	23	23	7	15	19	30
[5 – 15)	no	1228	1415	808	1025	1036	1213
	yes	90	82	41	61	77	101
[15 – 30)	no	1327	1449	975	1029	1022	1246
	yes	164	182	103	118	121	154
[30 – 45)	no	1254	1209	788	761	785	914
	yes	297	304	211	218	234	293
[45 – 60)	no	854	873	578	607	592	769
	yes	672	648	461	435	416	568
[60 – 70)	no	362	372	220	203	168	211
	yes	703	908	504	464	459	545
[70+)	no	239	170	145	121	150	181
	yes	598	463	399	489	499	657
**Total**		8410	8785	5609	6051	6062	7456

Supplementary Table 6: Number of survey participants in the different aggregated periods by age group and chronic disease.

Acute disease

Age Group	Acute disease	1st IP	2nd W	3rd W	2nd IP	4th W	5th W
[0 – 5)	no	618	696	371	511	475	586
	yes	4	14	5	8	28	18
[5 – 15)	no	1303	1472	836	1069	1076	1283
	yes	15	25	13	16	37	31
[15 – 30)	no	1458	1585	1051	1097	1083	1348
	yes	33	46	27	50	60	52
[30 – 45)	no	1503	1456	957	923	948	1152
	yes	48	57	42	56	71	55
[45 – 60)	no	1424	1405	969	972	931	1223
	yes	102	116	70	70	77	114
[60 – 70)	no	980	1135	652	611	571	672
	yes	85	145	72	56	56	84
[70+)	no	767	569	501	551	593	756
	yes	70	64	43	59	56	82
**Total**		8410	8785	5609	6049	6062	7456

Supplementary Table 7: Number of survey participants in the different aggregated periods by age group and acute disease.

Smoking

Age Group	Smoking	1st IP	2nd W	3rd W	2nd IP	4th W	5th W
[15 − 30)	no	569	549	370	353	357	419
	yes	452	505	324	349	340	444
[30 − 45)	no	965	964	628	600	620	768
	yes	586	549	371	379	399	439
[45 − 60)	no	879	913	674	646	633	864
	yes	647	608	365	396	375	473
[60 − 70)	no	711	839	458	431	404	500
	yes	354	441	266	236	223	256
[70+)	no	724	527	453	520	531	688
	yes	113	106	91	90	118	150
**Total**		6000	6001	4000	4000	4000	5001

Supplementary Table 8: Number of survey participants in the different aggregated periods by age group and smoking behaviour.

1.3 Data pre-processing

All the analyses on the number of contacts have been performed after having deleted the outliers at the 98% percentile with respect to the period of interest.

All the results presented in this work have been computed by accounting for each participant according to its weight. The weight has been provided by the survey company as described in the MM section of the main text. In the raking procedure for the creation of the weights, in each iteration weights were calculated for one dimension to align the sample total with the population total by the given dimension (weighting variable): first for the first dimension, then for the second dimension, and so on. These iterations continued across all dimensions until the final weights made the distributions of all weighting variables the same as the population distribution (within an acceptable margin of error). [5] Weighting dimensions included gender, age, education and domicile. As a result of the procedure, each respondent was assigned with a weight, indicating whether they should have higher, or lower relative influence in the analysis. For the calculation of the weights, SPSS's SPSS_RAKE function was used [5].

2 Robustness checks on the statistical model

In this section, we provide all the results of the additional statistical analysis that we performed in order to support the findings presented in the Result section of the main text.

2.1 Model on contacts

2.1.1 Accounting for the high number of zeros

All contacts Due to the implementation of NPIs (lockdowns and curfews) throughout the pandemic, people were forced, when possible, to reduce or completely reset their number of contacts. Thus, when analysing the distribution of the number of contacts we found a high presence of zeros.

To test the robustness of the results of the negative binomial regression model (*nb*) to this zero-inflated mechanism we implemented two additional models:

1. After having excluded the observations where the number of contacts was zero we re-run the *negative binomial regression model* on the non-zero number of contacts ($nb_{\text{ncontacts}>0}$).

$$\mu_i = \alpha + \beta_1 \text{age\_group}_i + \beta_2 X_i + \beta_3 \text{age\_group}_i * X_i + \epsilon_i \quad (1)$$

2. We modelled the probability to have at least one contact using a *logistic regression model* (*logit*).

$$\log P_i(\text{anycontact} = 1) = \alpha + \beta_1 \text{age\_group}_i + \beta_2 X_i + \beta_3 \text{age\_group}_i * X_i + \epsilon_i \quad (2)$$

where age_group_i is the age class of i; X_i is the variable of interest (e.g., education, income etc.), $\text{age\_group}_i * X_i$ is the interaction term of the age group and the variable of interest, and ϵ_i is the error term. In model (1) given μ_i, we define $\lambda_i = \exp(\mu_i)$ to be the expected number of contacts for respondent i. While in model (2) $P_i(\text{anycontact} = 1)$ indicate the probability for respondent i to have at least one contact.

By applying the same methodology as explained in the Method section we computed the *max confidence level* by age for each of the variables and period considered in the analysis for model (1) and (2). Fig 1 shows the results of the three models that we implemented. Interestingly, we can see that the same qualitative patterns result from both the negative binomial models, that is if we include the observations where the number of contacts is 0 (*nb*), or we discard it ($nb_{\text{ncontacts}>0}$) (Fig. 1a, b). In particular, although the *maximum confidence level* computed with these models differ in terms of variability, education, employment and income seem to remain the most significant dimensions in terms of explaining differences in contact numbers among subgroups of the population. The logistic regression model (*logit*) (Fig. 1c), shows similar results regarding the education and employment situation while it indicates that the other variables analyzed are not determining if individuals have contacts or not.

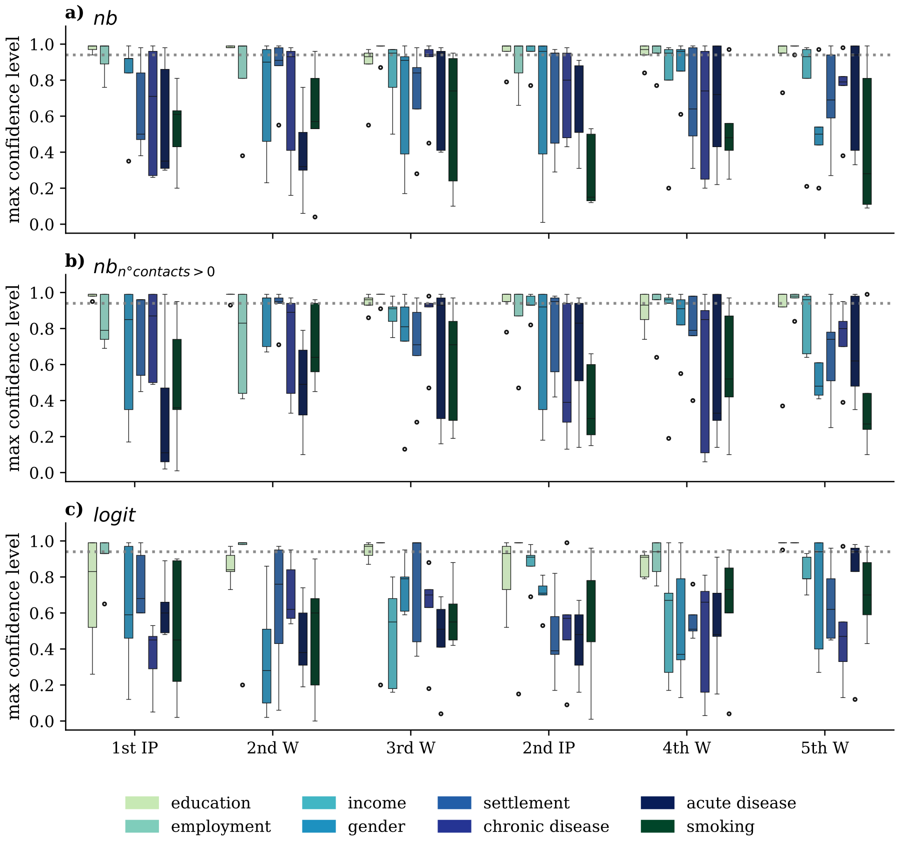

Supplementary Figure 1: Box-plot of the maximum confidence level at which the effect of the different categories of the variable on the dependent variable of contacts becomes significantly different. The dispersion of the box plot refers to the variation of this value over different age groups. Results are shown for *education level, employment situation, income level, gender, settlement, chronic disease, acute disease, and smoking behaviour*. Results for the three models implemented *nb* (negative binomial) (a), $nb_{\text{ncontacts}>0}$ (negative binomial on non-zeros number of contact) (b), and *logit* (logistic regression) (c).

Community contacts Furthermore, we also investigated the contacts happening exclusively in the community layer, excluding the ones happening at work. Here we present the results of the statistical analysis on the number of contacts in the community layer. Particularly, in Fig. 2 we show the results of the three models we are implementing: 1. negative binomial regression (*nb*), 2. negative binomial regression only on positive observations of the number of contacts ($nb_{\text{ncontacts}>0}$), and 3. logistic regression to model the probability of having at least one contact (*logit*). In all these models the dependent variable (y_i) refers to the community-level contacts of the individuals. Results are similar to the ones obtained considering all the types of contacts together. Indeed, also in this case results indicate that education, employment and income are the most significant dimensions in terms of explaining differences in contact numbers in the community layer among subgroups of the population. Also the logistic regression model (*logit*) (Fig. 2*c*), shows similar results indicating that only education and employment are significant in determining if individuals have contacts or not in the community layer.

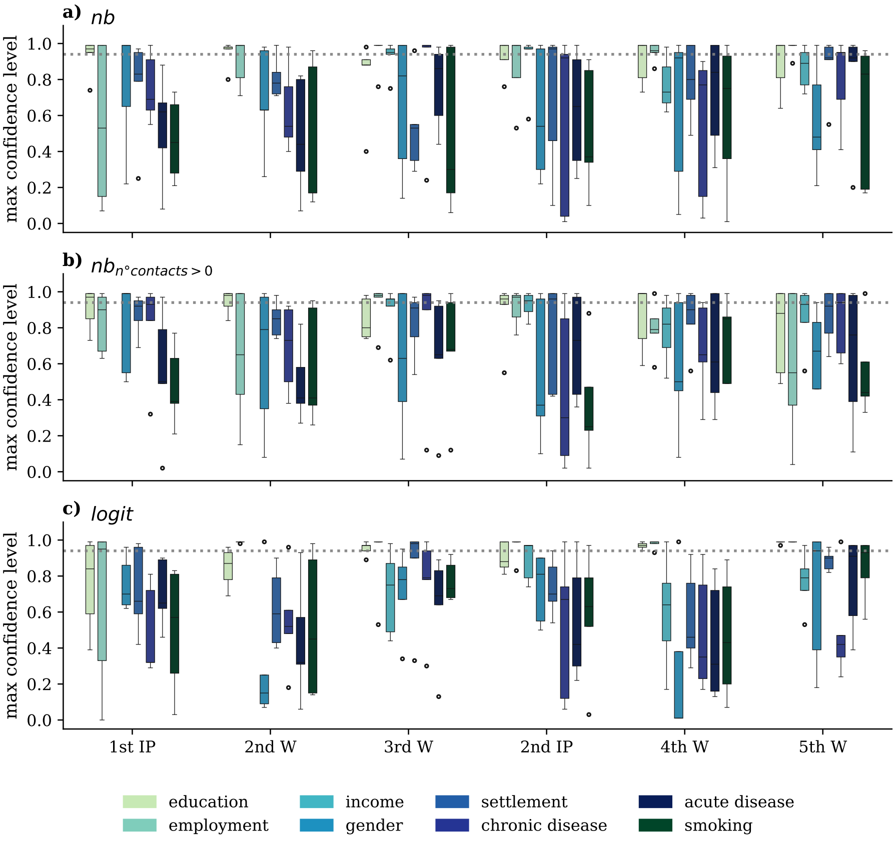

Supplementary Figure 2: Box-plot of the maximum confidence level at which the effect of the different categories of the variable on the dependent variable of contacts becomes significantly different. The dispersion of the box plot refers to the variation of this value over different age groups. Results are shown for *education level, employment situation, income level, gender, settlement, chronic disease, acute disease, and smoking behaviour.* Results for the three models implemented *nb* (negative binomial) (*a*), $nb_{\text{ncontacts}>0}$ (negative binomial on non-zeros number of contact) (*b*), and *logit* (logistic regression) (*c*).

2.1.2 Accounting for the effect of control variables

NPIs and vaccination behaviour Although, the data were aggregated to enhance homogeneity in implemented non-pharmaceutical interventions (NPIs), yet minor variations in restrictions persist within certain periods (Fig. 3a). In addition, we account for the interplay of vaccination behaviour and contacts which can be quite important in a dynamic situation when vaccination strategies (Fig. 3b) are implemented over an epidemic crisis such as the one of COVID-19 [17] Thus, we have incorporated two additional controls variable to the statistical model:

- the *Oxford Stringency Index (OSI)*. This index is a composite measure that aims to quantify the strictness of the government's response to the COVID-19 pandemic. It is computed as a composite measure of nine of nine metrics: school closures; workplace closures; cancellation of public events; restrictions on public gatherings; closures of public transport; stay-at-home requirements; public information campaigns; restrictions on internal movements; and international travel controls. [10]
- a dummy variable that indicates if an individual is vaccinated (vax_i), this variable is available only from the $3rd$ wave on.

The results are shown in Fig. 4 and Fig. 5 respectively for the total number of contacts and the community ones. As we can observe the results are very similar to the one shown above, thus they bring to the same qualitative conclusions.

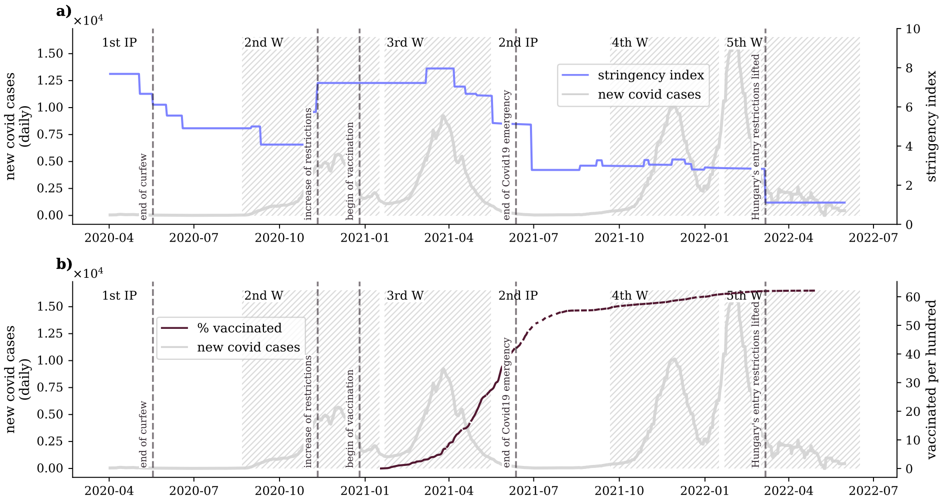

Supplementary Figure 3: **a)** *Left axis*: number of new daily COVID-19 cases in Hungary from 2020/04 to 2022/07. *Right axis*: Stringency Index. **b)** *Left axis*: number of new daily COVID-19 cases in Hungary from 2020/04 to 2022/07. *Right axis*: Percent of vaccinated people in the Hungarian population. Source: our-word-in-data. In both panels, the white and grey areas delimit the periods that have been aggregated in the analysis: two interim periods (IPs) (white areas) and four epidemic waves (W) (grey dashed areas).

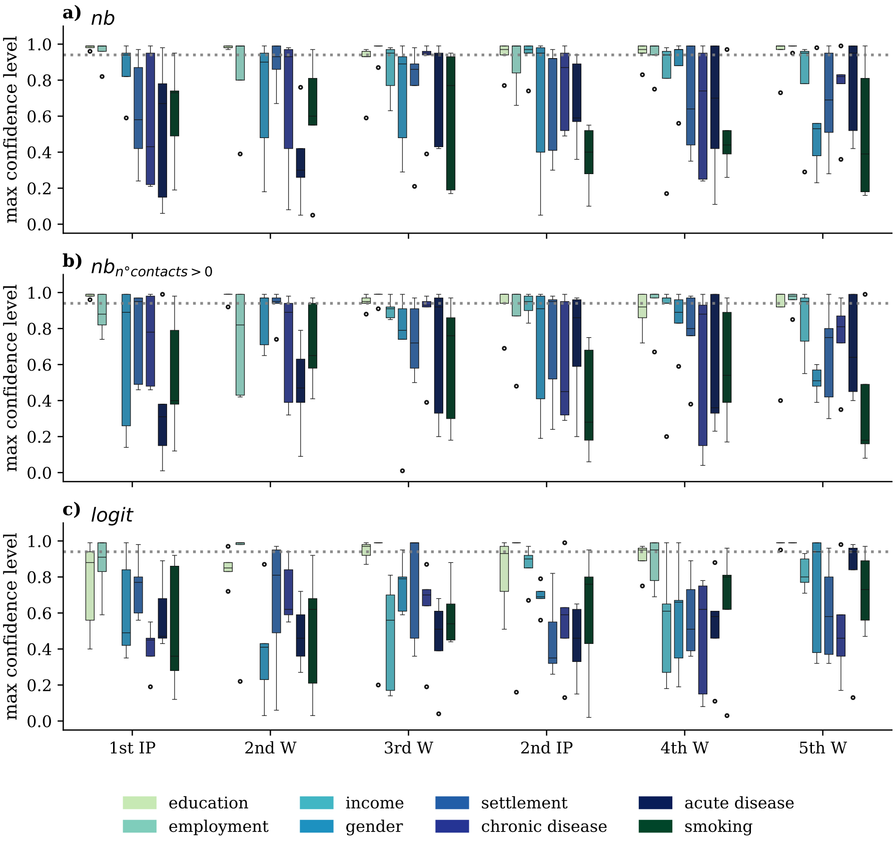

Supplementary Figure 4: Box-plot of the maximum confidence level at which the effect of the different categories of the variable on the dependent variable of contacts becomes significantly different. The dispersion of the box plot refers to the variation of this value over different age groups. Results are shown for *education level, employment situation, income level, gender, settlement, chronic disease, acute disease, and smoking behaviour*. Results for the three models implemented *nb* (negative binomial) (*a*), $nb_{\text{ncontacts}>0}$ (negative binomial on non-zeros number of contact) (*b*), and *logit* (logistic regression) (*c*).

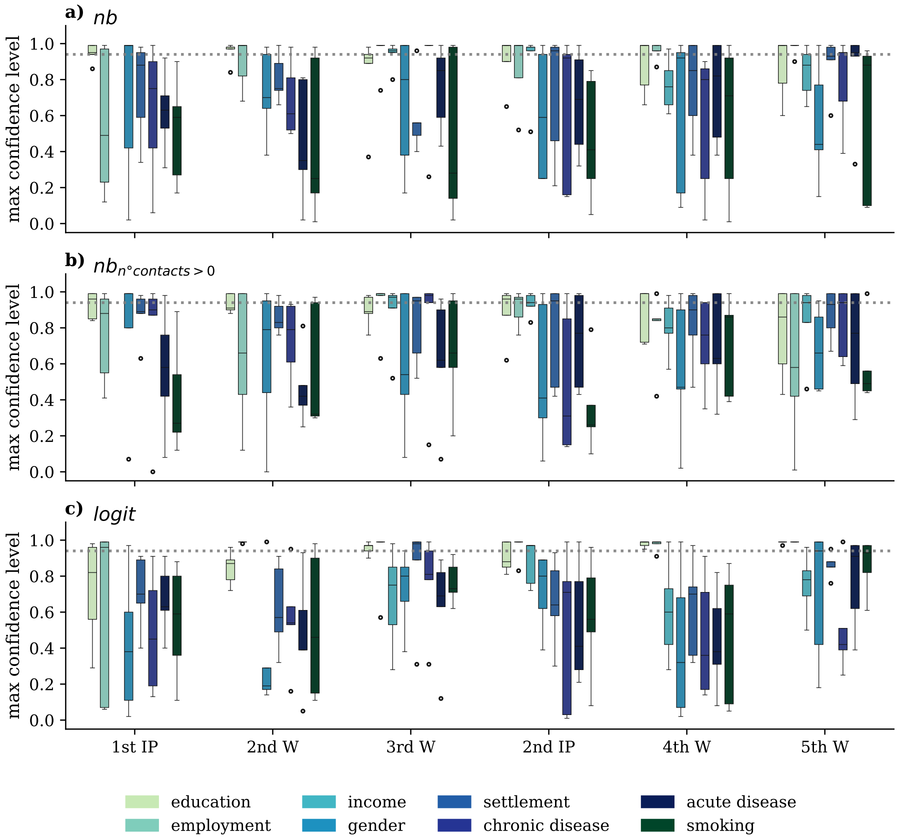

Supplementary Figure 5: Box-plot of the maximum confidence level at which the effect of the different categories of the variable on the dependent variable of contacts becomes significantly different. The dispersion of the box plot refers to the variation of this value over different age groups. Results are shown for *education level, employment situation, income level, gender, settlement, chronic disease, acute disease, and smoking behaviour*. Results for the three models implemented *nb* (negative binomial) (a), $nb_{\text{ncontacts}>0}$ (negative binomial on non-zeros number of contact) (b), and *logit* (logistic regression) (c).

Other control variables To enhance the statistical robustness of our findings, additional analyses were conducted by incorporating supplementary control variables into the model. Specifically, for each variable of interest in the model, all the other variables were included as controls, in addition to the one presented above (OSI and vax_i). The outcomes are shown in Fig. 6 for total contacts and Fig. 7 for community contacts. Notably, the results closely align with those presented earlier, reinforcing the robustness of our qualitative conclusions.

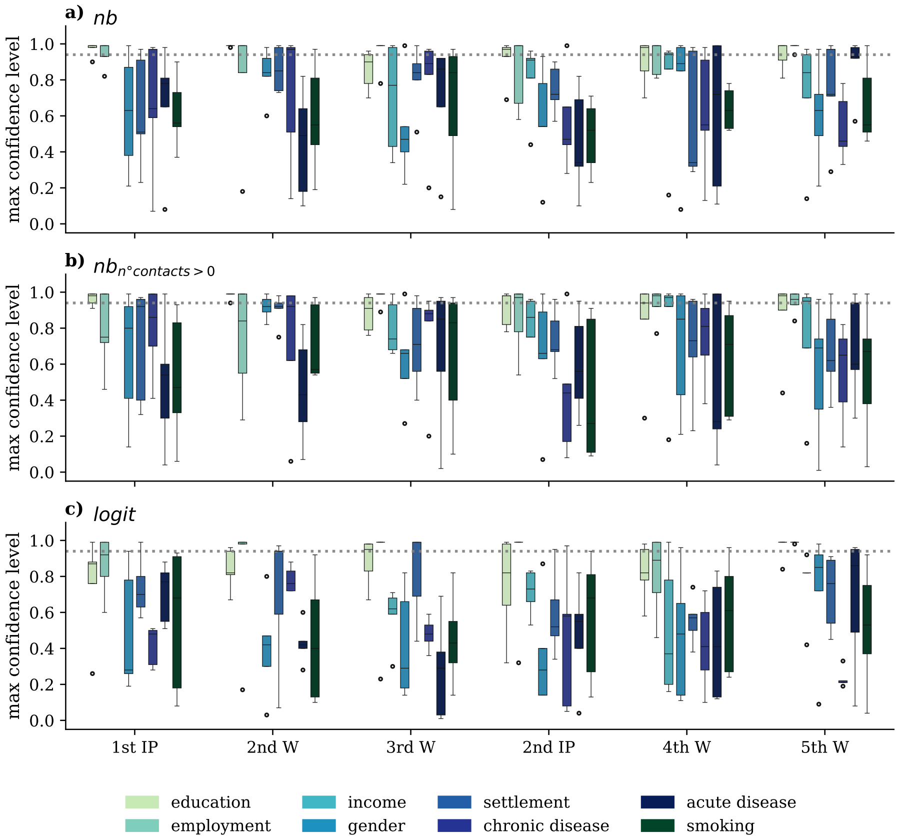

Supplementary Figure 6: Box-plot of the maximum confidence level at which the effect of the different categories of the variable on the dependent variable of contacts becomes significantly different. The dispersion of the box plot refers to the variation of this value over different age groups. Results are shown for *education level, employment situation, income level, gender, settlement, chronic disease, acute disease, and smoking behaviour.* Results for the three models implemented *nb* (negative binomial) (a), $nb_{\text{ncontacts}>0}$ (negative binomial on non-zeros number of contact) (b), and *logit* (logistic regression) (c).

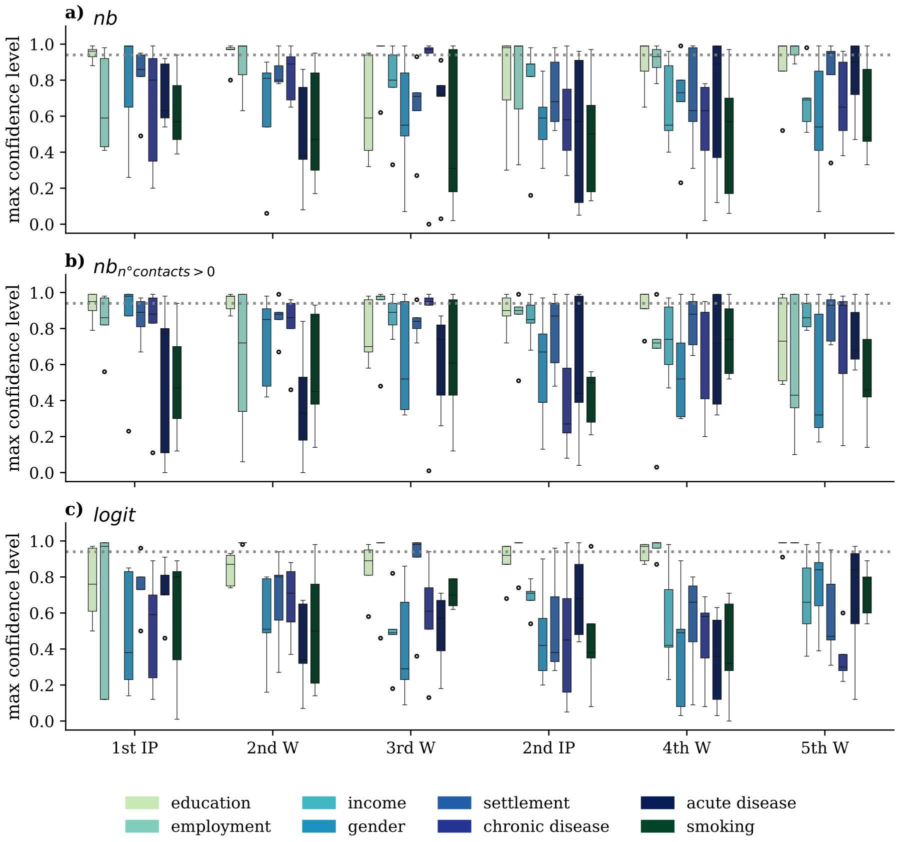

Supplementary Figure 7: Box-plot of the maximum confidence level at which the effect of the different categories of the variable on the dependent variable of contacts becomes significantly different. The dispersion of the box plot refers to the variation of this value over different age groups. Results are shown for *education level, employment situation, income level, gender, settlement, chronic disease, acute disease, and smoking behaviour*. Results for the three models implemented *nb* (negative binomial) (a), $nb_{\text{ncontacts}>0}$ (negative binomial on non-zeros number of contact) (b), and *logit* (logistic regression) (c).

2.2 Model on vaccination

We model the probability of getting vaccinated against COVID-19 using a logistic regression model. Namely, we model the probability of getting vaccinated for respondent i as defined in Eq. (3):

$$\log P_i(\text{vax} = 1) = \alpha + \beta_1 rmage\_group_i + \beta_2 X_i + \beta_3 \text{age\_group}_i * X_i + \epsilon_i \qquad (3)$$

where age_group_i is the age class of i; X_i is the variable of interest (e.g., education, income etc.), $\text{age\_group}_i * X_i$ is the interaction term of age group and the variable of interest, and ϵ_i is the error term.

By applying the same methodology as explained in the MM section we computed the *max confidence level* by age for each of the variables and period considered in the analysis (Fig. 8)

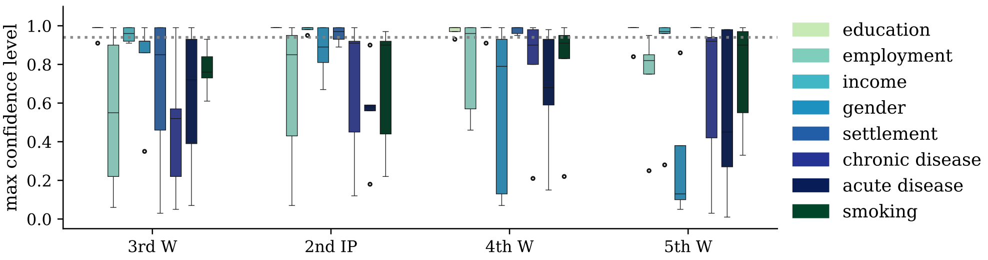

Supplementary Figure 8: Box-plot of the maximum confidence level at which the effect of the different categories of the variable on vaccination become significantly different. The dispersion of the box plot refers to the variation of this value over different age groups. Results are shown for *education level, employment situation, income level, gender, settlement, chronic disease, acute disease, and smoking behaviour.*

2.2.1 Accounting for the effect of NPIs

As we did for the model of contacts, to incorporate the impact of non-pharmaceutical interventions (NPIs) on vaccination uptake, we have incorporated an additional control variable to the statistical model: the *Oxford Stringency Index (OSI)* [10]. The results are shown in Fig. 9. As we can observe the results are very similar to the one shown above, thus they bring to the same qualitative conclusions.

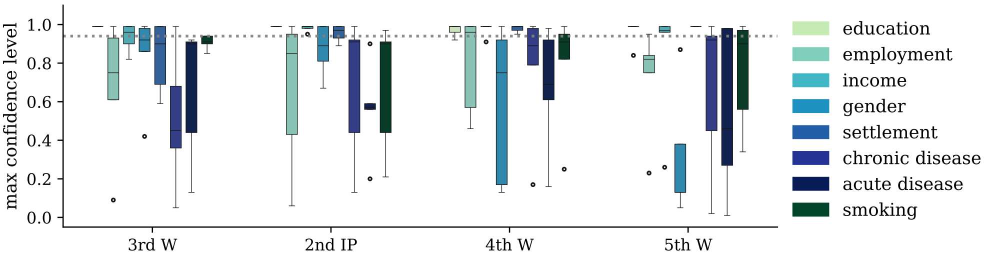

Supplementary Figure 9: Box-plot of the maximum confidence level at which the effect of the different categories of the variable on vaccination becomes significantly different. The dispersion of the box plot refers to the variation of this value over different age groups. Results are shown for *education level, employment situation, income level, gender, settlement, chronic disease, acute disease, and smoking behaviour.*

3 Bootstrapping

In this study, we employ bootstrapping sampling technique to assess the uncertainty of the estimation of contacts. We used bootstrapping for the estimation of average contact numbers by the different levels of the social-demographic variables, and also for the estimation of the decoupled age contact matrices In particular, the bootstrapping method involves resampling with replacement from the original dataset, specifically focusing on a subset of the data stratified simultaneously by the given variable of interest (*employment, education level, income level and settlement*) and in case of matrices by age as well. By iteratively drawing multiple samples from each subset, we create a distribution of the average number of contacts by the levels of the variable of interest (and in the case of matrices by age as well), allowing us to quantify the uncertainty in the estimate and derive confidence intervals. All the results are conveyed as the median of 1000 bootstrapped samples, accompanied by their interquartile range (IQR)

4 Contacts

4.1 Average number of contacts in the community and in the work layer by sub-groups

In the main text, we show the evolution of contacts over time decoupled by education level and employment. For completeness, here we report the same figures for income level Fig 10-a,c and settlement Fig 10-b,d. Looking at the community contacts we can observe that there is a clear rank among the income levels in their number of contacts, with high-income individuals having the highest number of contacts and low-income individuals having the lowest. The same is arguable for the individuals living in the capital, which appear to be the most active in the community layer, while, individuals living in rural areas are the less active. To what concern the contacts at work, we can clearly observe that high-income individuals were the ones who would better adapt to the epidemiological situation, while mid and low income maintained a fairly stable number of contacts over time at the workplace. Particularly, they reported a higher number of contacts at the workplace during the COVID-19 waves. A similar conclusion can be drawn when we decoupled individuals according to their settlement. In this case, individuals living in rural areas appear to be the most active in the workplace while the ones living in the capital tend to have a lower number of interactions in the workplace.

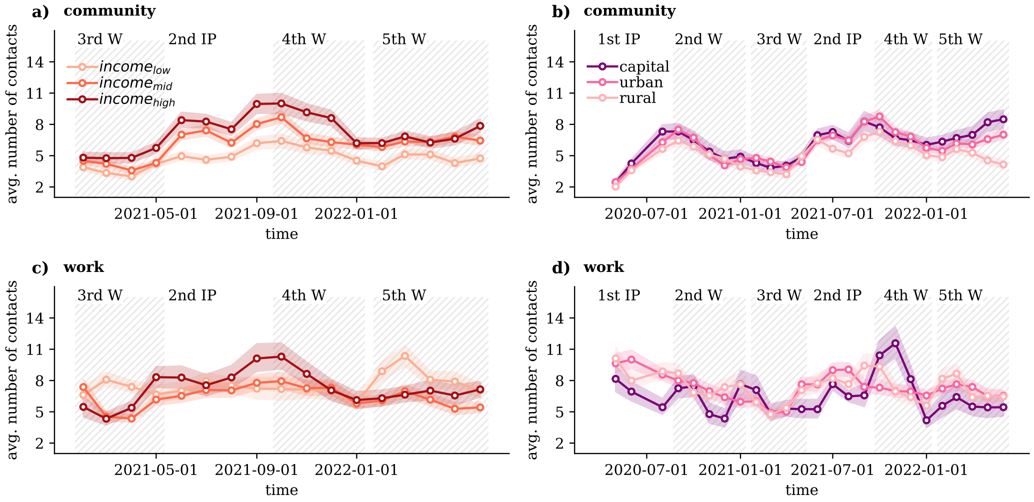

Supplementary Figure 10: **(a-b)** Smoothed average number of contacts in the community layer by different income levels *a*) and settlement types *b*). **(c-d)** Smoothed average number of contacts at the workplace by different income levels *c*) and settlement types *d*). All the values are shown as the median and the IQR of 1000 bootstrapped samples.

4.1.1 Relative contact variation

Here we present the relative number of contacts categorized by employment situation and education level (Fig. 11), as well as by income level and settlement (Fig. 12). Namely, in each month, the relative number of contacts of each subgroup is computed as the ratio between the average number of contacts for each subgroup and the average of the total number of contacts in the population.

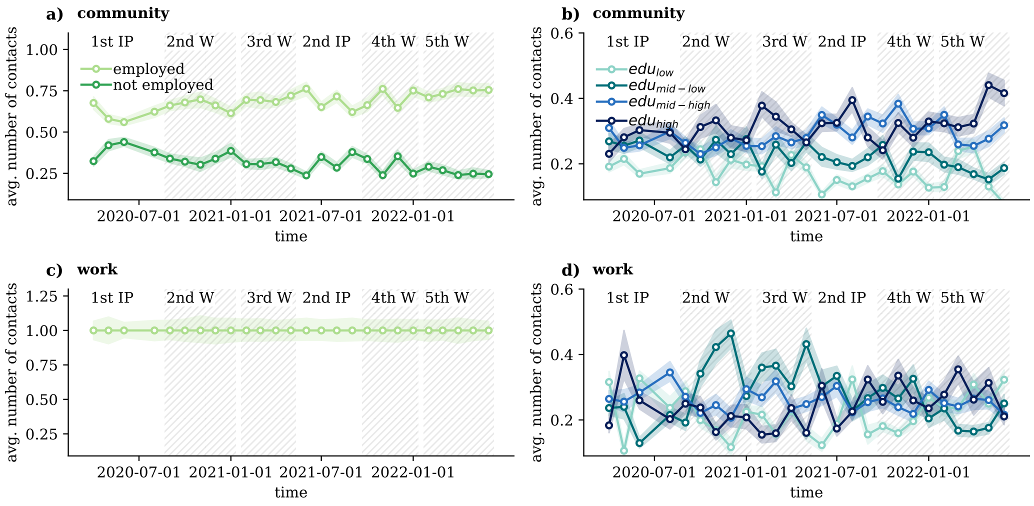

Supplementary Figure 11: **(a-b)** Relative smoothed average number of contacts in the community layer by different employment situation *a*) and education levels *b*). **(c-d)** Relative smoothed average number of contacts at the workplace by different employment situation *c*) and education level *d*). All the values are shown as the median and the IQ of 1.000 bootstrapped samples.

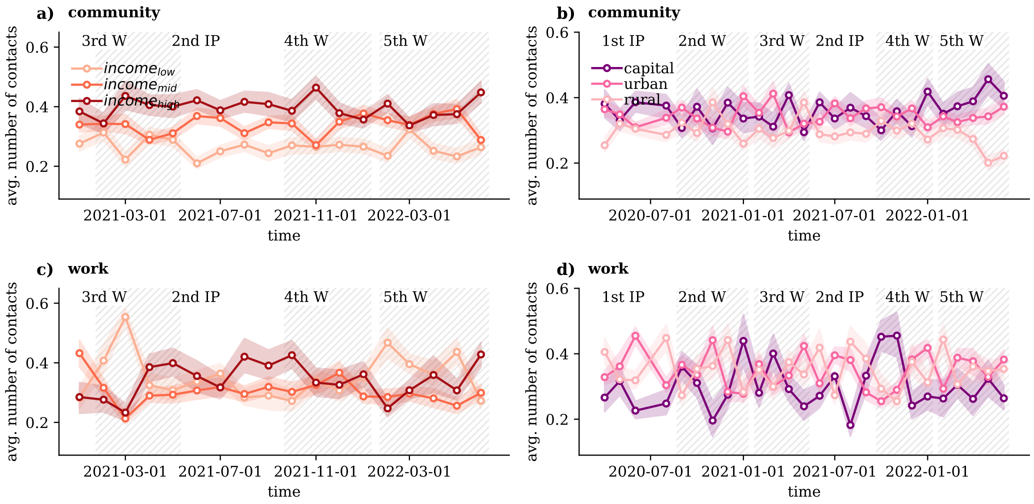

Supplementary Figure 12: **(a-b)** Relative smoothed average number of contacts in the community layer by different income levels *a*) and settlement types *b*). **(c-d)** Relative smoothed average number of contacts at the workplace by different income levels *c*) and settlement types *d*). All the values are shown as the median and the IQR of 1000 bootstrapped samples.

4.2 Average number of contacts in the community layer by sub-groups and age groups

To show the robustness of our findings over different age groups in Fig. 13 we report the evolution of community contacts over time decoupled by education level, employment, income level and settlement. While the correlation with age influences the magnitude of differences among the examined sub-groups, the conclusion discussed in the main text appears to be still valid.

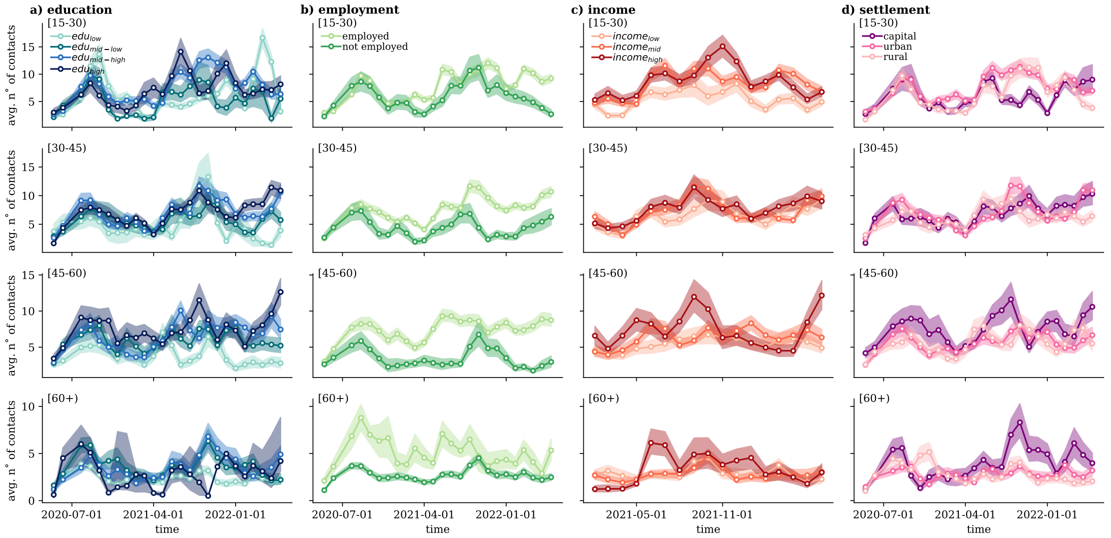

Supplementary Figure 13: Average number of contacts in the community layer by different age groups*(rows)* and sub-groups *(columns*: education levels *a*), employment situation *b*, income levels *c*) and settlement *d*). All the values are shown as the median and the IQR of 1000 bootstrapped samples.

4.3 Contact Matrices

4.3.1 Symmetrization of the decoupled contact matrices

The nature of social interaction poses the crucial constraint of *reciprocity* to the calculation of the age-contact matrix, that is, the total number of contacts that individuals in age-group i with individuals in age-group j must be equal to the total number of contacts that individuals in age-group j form with individuals in age-group i, such that: $C_{ij}N_i = C_{ji}N_j$. However, due to limitations in the survey and errors in the reporting process, age contact matrices will not fulfil reciprocity perfectly and symmetrization procedures are needed to impose such conditions [16, 6, 1]. In the context of traditional age contact matrices, these are typically straightforward. Usually, the age contact matrix of the total number of contacts is symmetrized as follows:

$$C_{ij}^{sym} = \frac{C_{ij}N_i + C_{ji}N_j}{N_i 2_i} \tag{4}$$

However, the symmetrization of decoupled age-contact matrices poses a non-trivial challenge. Given a set of *decoupled* age-contact matrices along a given dimension d the reciprocity condition becomes: $\sum_d C_{d,ij}N_{di} = \sum_d C_{ji}N_{d,ij}$. Thus, the difficulty in symmetrization arises from the fact that we can not compute the transpose of a decoupled

age-contact matrix. This is because the matrix is stratified only along the columns i.e. the participant's subgroup of appartenence.

To address this challenge, we implemented the following approach. For each of the decoupled age-contact matrices ($C_{d,ij}$) we compute a corresponding scaling matrices ($B_{d,ij}$). These matrices represent the proportions of contributions of each decoupled contact matrix to the aggregate one.

$$B_{d,ij} = C_{d,ij} N_{d,i} / C_{ij} N_i \tag{5}$$

Subsequently, akin to Equation (4), we computed the symmetric age-contact matrix at the aggregated level and we project back the symmetrized matrix (C_{ij}^{sym}) to the decoupled dimensions as follows:

$$C_{d,ij}^{sym} = \frac{C_{ij}^{sym} N_i \times B_{d,ij}}{N_{d,i}} \tag{6}$$

4.3.2 Derived contact matrices

For each of the periods considered in this study, we computed the age contact matrix C_{ij} considering the whole population as shown in Fig. 14. These are the matrices that have been fed to the *conventional* SEIR model. Instead for the *extended* SEIR model, we computed the decoupled contact matrices ($C_{di,j}$) considering different dimensions as well: (*i*) employment situation (Fig. 15), (*ii*) education level (Fig 16),(*iii*) settlement (Fig. 17), and (*iv*) income level (Fig. 18). For all the matrices we report the median of 1000 bootstrapped samples. All the matrices have been computed considering the contact at work, in the community and in the household. A detailed explanation of the computation of such matrices is provided in the Method section.

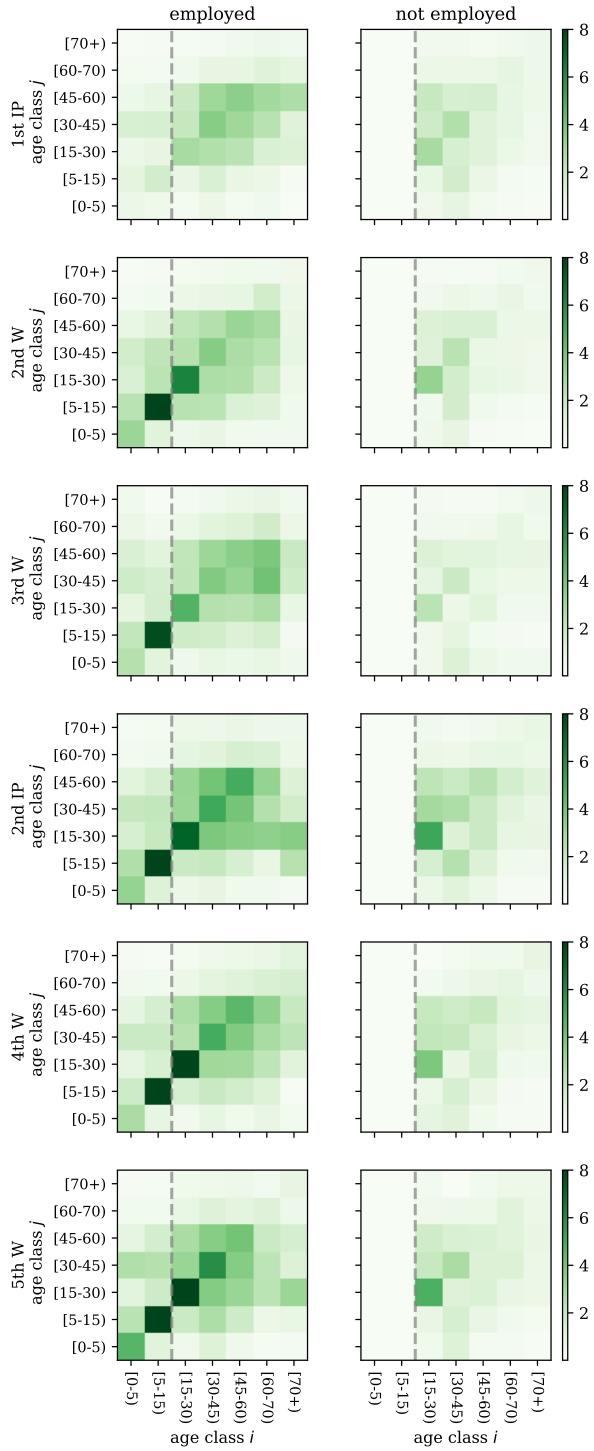

Supplementary Figure 14: Age contact matrices (C_{ij}) for different periods.

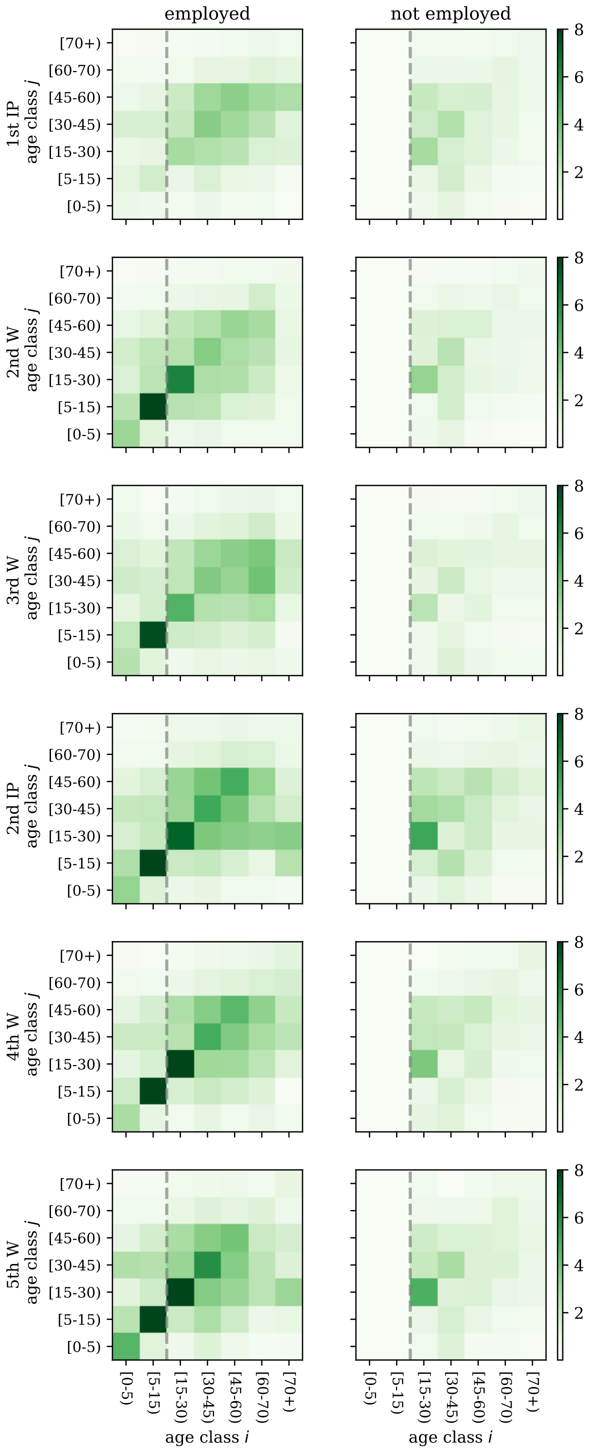

Supplementary Figure 15: Age contact matrices decoupled by employment situation ($C_{\mathrm{employment}i,j}$) for different periods.

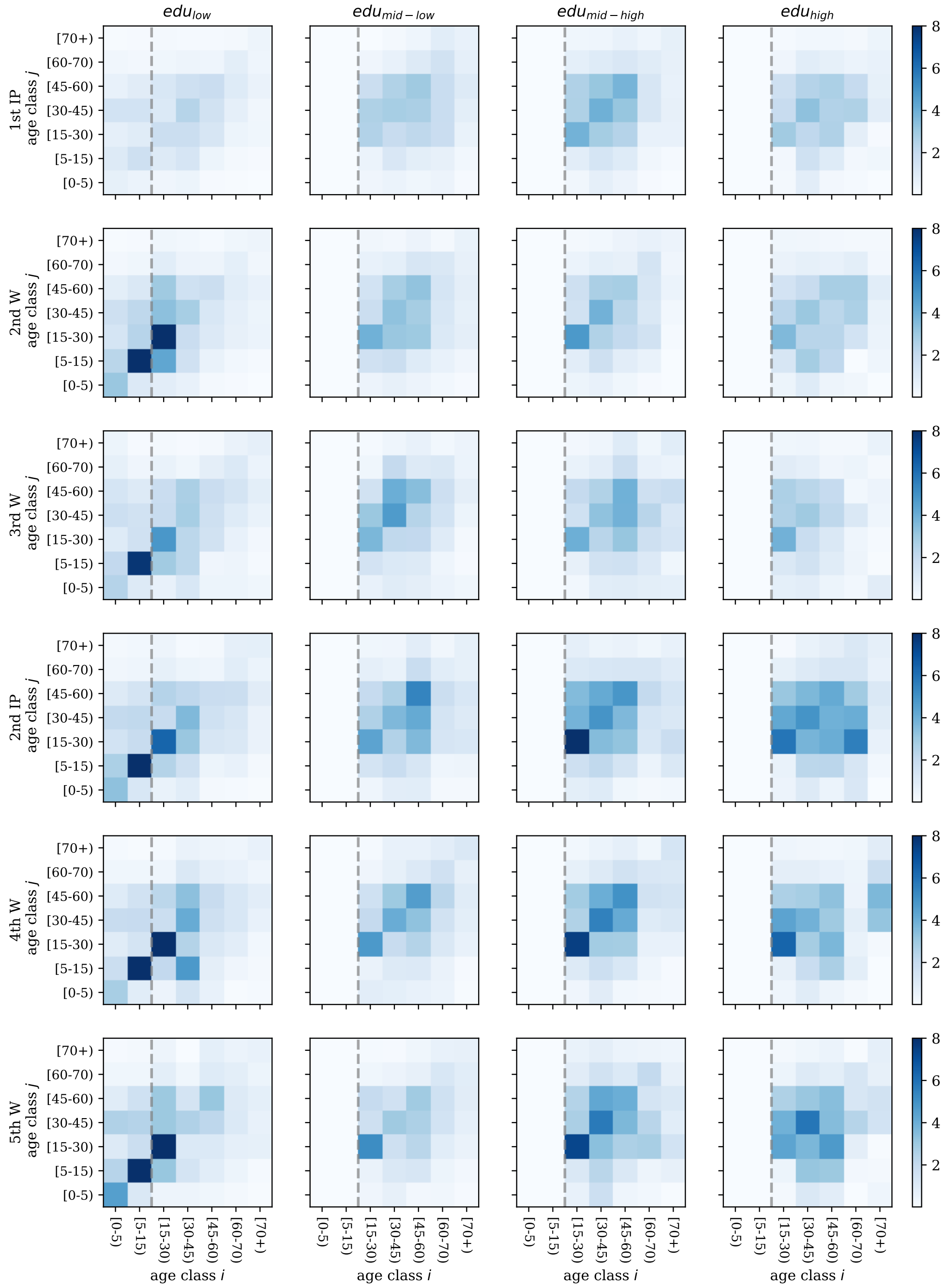

Supplementary Figure 16: Age contact matrices decoupled by education level ($C_{\text{education}i,j}$) for different periods.

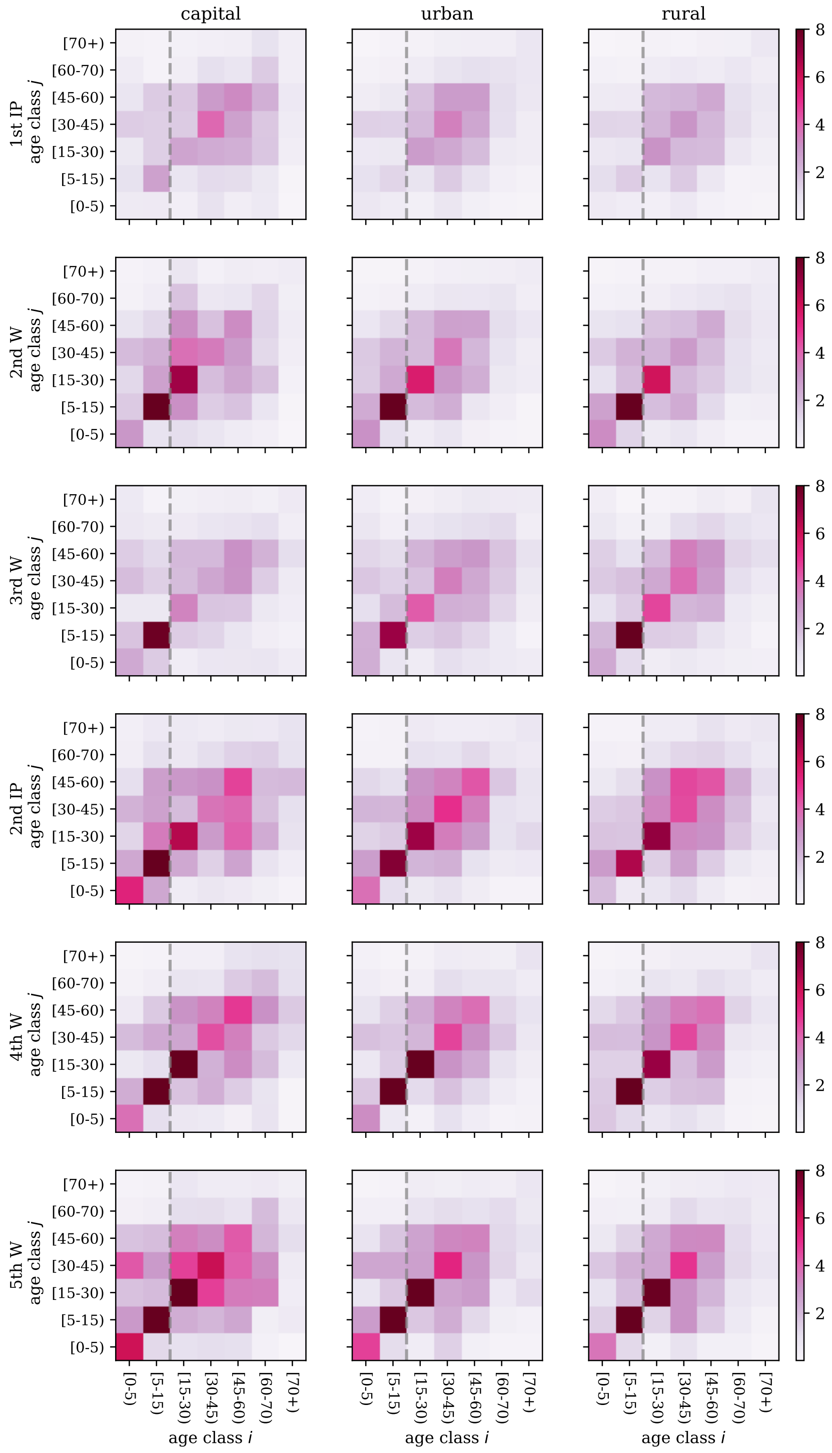

Supplementary Figure 17: Age contact matrices decoupled by settlement ($C_{\text{settlement}i,j}$) for different periods.

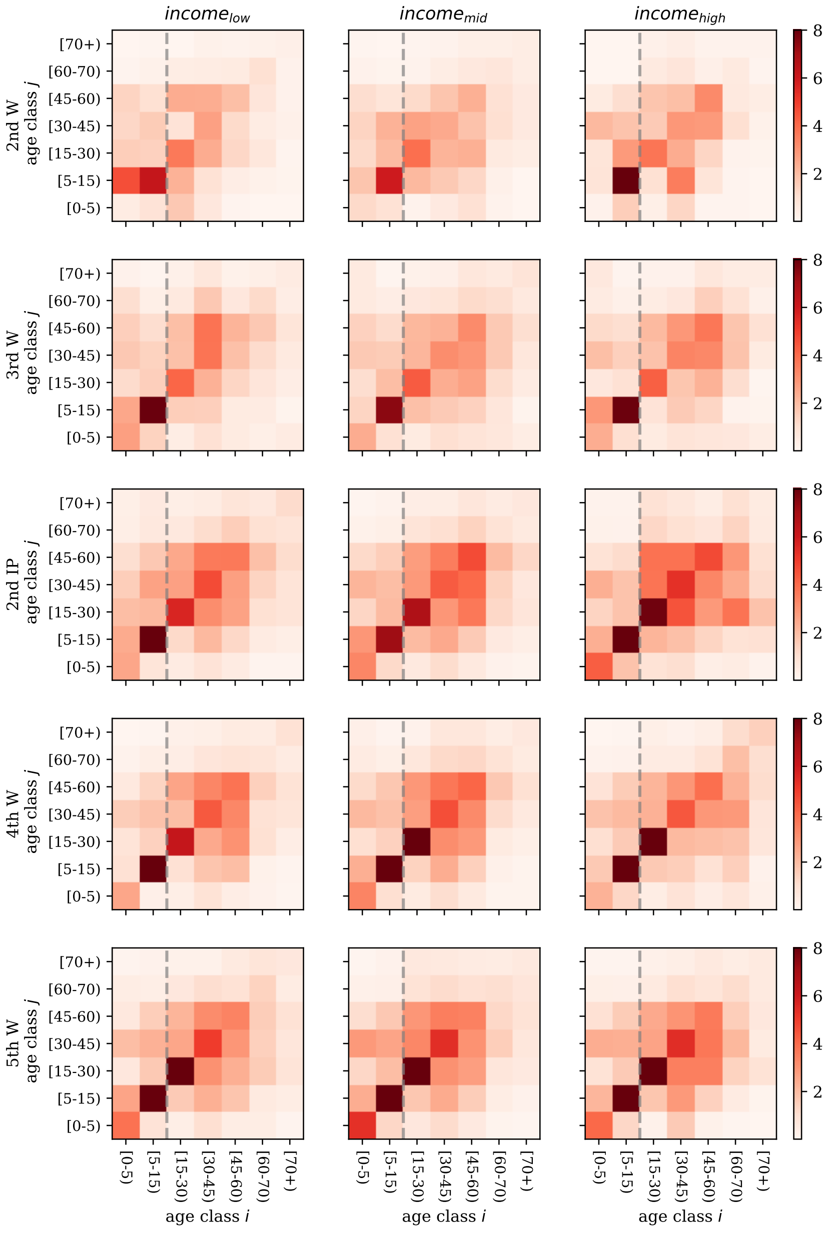

Supplementary Figure 18: Age contact matrices decoupled by income level ($C_{\text{income}i,j}$) for different periods.

5 Vaccination uptake

Here we show the probability of getting vaccinated against COVID-19 given age and another dimension of interest. Namely, we consider (i) employment situation, (ii) education level,(iii) settlement, and (iv) income level. From Fig. 19 we can observe that privileged groups of the population tend to have higher vaccination uptake across all age groups. This finding is consistent over the four different periods considered in the analysis.

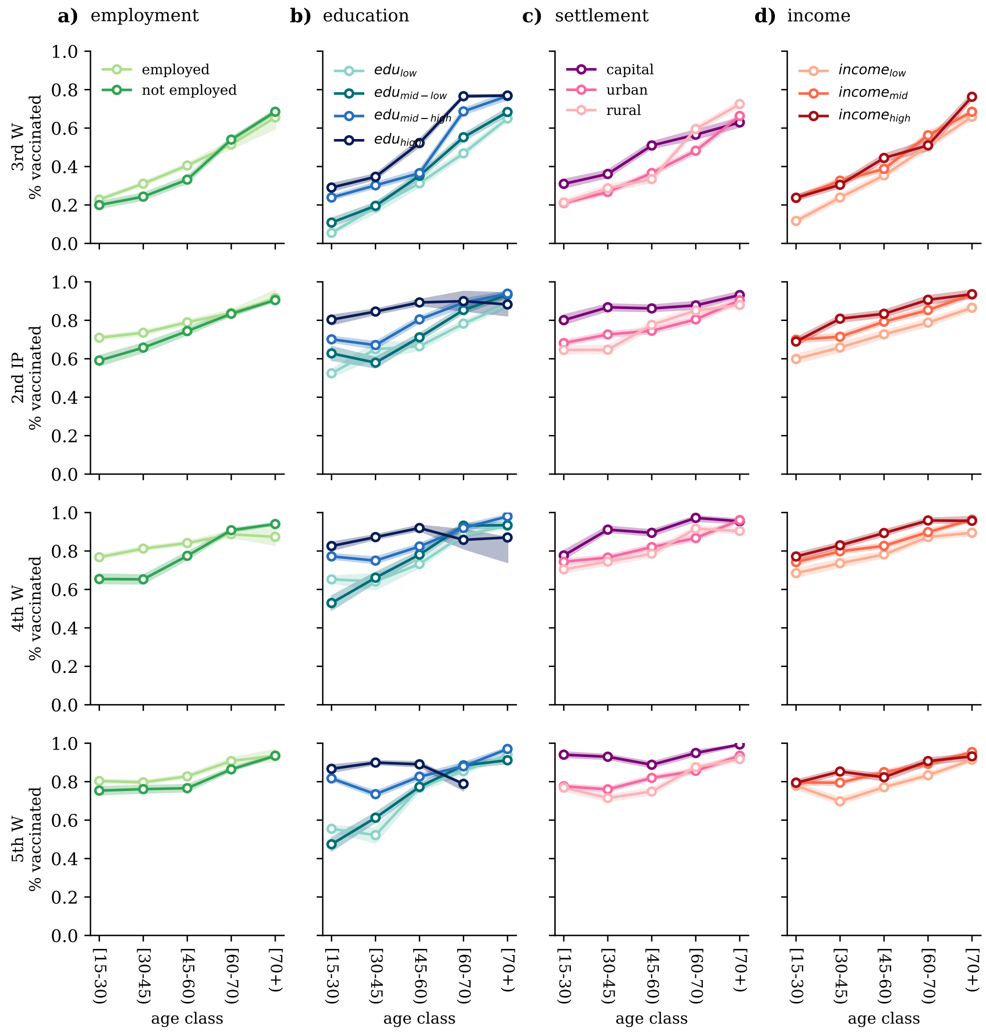

Supplementary Figure 19: Probability of getting vaccinated given age and (i) employment situation, (ii) education level,(iii) settlement, and (iv) income level.All the values are shown as the median and the IQR of 1000 bootstrapped samples.

6 Epidemic models

In this section, we report the ODE's equation of the (i) *conventional* age-stratified SEIRD model and, (i) *extended* SEIRD where beyond the age stratification we can differentiate the population along other dimensions of interest $(\bar{d})$. Specifically, the reported equations for the *extended* SEIRD model account also for vaccination.

6.1 *Conventional* age-stratified SEIRD

Let's consider an infectious disease that can be described with a Susceptible-Exposed-Infected-Recovered-Death model [7]. The epidemic dynamic is encoded in the set of differential equations in Eq. (7). Where i indicates the age group of the ego, j indicates the age group of the alter, β is the probability of transmission given a contact, ϵ is the rate at which individuals become infectious, μ is the recovery rate, C_{ij} is the age contact matrix, and IFR_i is the infection fatality rate by age group.

$$\begin{array}{rcl} \dot{S}_i & = & -\lambda_i S_i \\ \dot{E}_i & = & \lambda S_i - \epsilon E_i \\ \dot{I}_i & = & \epsilon E_i - \mu I_i \\ \dot{R}_i & = & \mu I_i \\ \dot{D}_i & = & \mu IFR_i I_i \end{array} \tag{7}$$

The force of infection is defined as in eq. (8)

$$\lambda_i = \beta \sum_j \frac{C_{ij}}{N_j} I_j \tag{8}$$

6.2 *Extended SEIRD* with vaccination

We extend the *conventional SEIRD* model to account for different vaccination uptake of different groups of the population. Namely, each of the compartments is now considered separately for vaccinated and unvaccinated individuals. We define as g_1 and g_2 the efficiency of the vaccination respectively against infection and against death. In addition, we consider the delay in the official registrations of deaths by adding a new compartment Da and a delay of Δ^{-1} days. The equations of the model are presented in equation (9).

$$\begin{array}{rcl} \dot{S}_{\bar{d},i} & = & -\lambda_{\bar{d},i} S_{\bar{d},i} \\ \dot{Sv}_{\bar{d},i} & = & -(1-g_1)\lambda_{\bar{d},i} Sv_{\bar{d},i} \\ \dot{E}_{\bar{d},i} & = & \lambda_{\bar{d},i} S_{\bar{d},i} - \epsilon E_{\bar{d},i} \\ \dot{Ev}_{\bar{d},i} & = & (1-g_1)\lambda_{\bar{d},i} Sv_{\bar{d},i} - \epsilon Ev_{\bar{d},i} \\ \dot{I}_{\bar{d},i} & = & \epsilon E_{\bar{d},i} - \mu I_{\bar{d},i} \\ \dot{Iv}_{\bar{d},i} & = & \epsilon Ev_{\bar{d},i} - \mu Iv_{\bar{d},i} \\ \dot{R}_{\bar{d},i} & = & \mu(1-\mathrm{IFR}_i) I_{\bar{d},i} \\ \dot{Rv}_{\bar{d},i} & = & \mu(1-(1-g_2)\mathrm{IFR}_i) Iv_{\bar{d},i} \\ \dot{D}_{\bar{d},i} & = & \mu IFR_i I_{\bar{d},i} \\ \dot{Dv}_{\bar{d},i} & = & \mu(1-g_2)\mathrm{IFR}_i Iv_{\bar{d},i} \\ \dot{D}_{a\bar{d},i} & = & \Delta^{-1} \dot{D}_{\bar{d},i} \\ \dot{Dv}_{a\bar{d},i} & = & \Delta^{-1} \dot{Dv}_{\bar{d},i} \end{array} \tag{9}$$

The force of infection is defined as in eq. (10)

$$\lambda_{\bar{d},i}(t) = \beta \textstyle\sum_j \frac{C_{\bar{d},ij}}{N_j} [I_j + Iv_j] \tag{10}$$

7 Epidemic Simulations

We developed stochastic, discrete-time, compartmental models using chain binomial processes to simulate the transitions among compartments. Specifically, at each time step t, the model samples the number of individuals in group $(\bar{d}, i)$ and compartment X transitioning to compartment Y from $PrBin(X_{\bar{d},i}(t), p_{X_{\bar{d},i} \to Y_{\bar{d},i}}(t))$. Here, $p_{X_{\bar{d},i} \to Y_{\bar{d},i}}(t)$ represents the transition probability.

To illustrate this, let's consider the number of individuals in the group $(\bar{d}, i)$ and compartment S that at time t become exposed transiting to compartment E. Thus, the number of individuals in $S_{\bar{d},i}(t)$ getting exposed are extracted from a $PrBin(S_{\bar{d},i}(t), \lambda_{\bar{d},i}(t))$ where $\lambda_{\bar{d},i}(t)$ is the *force of infection*.

Furthermore, in order to account for the variability of contacts in our data, for each simulation that we run we use a static decoupled contact matrix that we compute from a bootstrapped sample of our data as described in Section 3. The decoupled contact matrices have been computed considering the contacts happening at work, in the community and with family members.

The model has been initialized by computing the population distributions from the MASZK data, while we set the Hungarian population size to 9.750.000.

While the epidemiological parameters are set to realistic values to closely simulate the characteristics of Covid-19. These values are retrieved from the literature. In particular, ϵ is set to 0.25; γ is set to 0.4 [4, 8, 2]. The transmission rate β is computed in each of the periods using the Next Generation Matrix approach [3] on the aggregate age-contact matrices corresponding to the periods analysed. We fixed $R0 = 2.5$ and we derived β using Eq. (11).

$$R_0 = \frac{\beta}{\mu} \rho(C_{ij}) \tag{11}$$

Where $\rho(C_{ij})$ is the spectral radius of the age contact matrix. In the simulations in which we introduce vaccination, we respectively set the $g_1 = 0.6$ and $g_2 = 0.8$ [15, 12]. The initial size of the epidemic is set to 5. All the results in the main text refer to the median over 1000 simulations of the model.

7.1 Impact of different contact patterns

In this section, we show the results of the *extended SEIR* model when differences in age contact matrices are considered for different sub-groups of the population. Particularly, we model age contact matrices differentiating individuals along their employment situation, education level, settlement and income level. For each of the dimensions considered, we run the *extended* SEIR model. We look at (i) how the prediction of this model differs from the *conventional SEIR* and (ii) how the attack rate differs for different subgroups as a result of their differences in contact patterns. Specifically, in Fig. 20 we show the difference between attack rate by age group as predicted by the *extended SEIR* model and the *conventional SEIR* model. The results are shown for each of the periods considered in the analysis. Again, as demonstrated in the main text, the *conventional SEIR* model tends to overestimate the attack rate by age group with respect to *extended SEIR* model, particularly when employment situation and education are taken into account.

In Fig. 21 we show the output of each of the *extended SEIR* models in terms of attack rate by age by differentiating along the subgroups taken into account. Results are shown

for each of the periods considered in the analysis.

As shown in the main text the analysis over the other periods confirms that employed and highly educated individuals happened to be the most infected groups in all age groups. When decoupling age contact matrices by settlement and income, although differences appear smaller between groups, high-income individuals and the ones living in the capital are more infected, particularly elderly ones with age 60+.

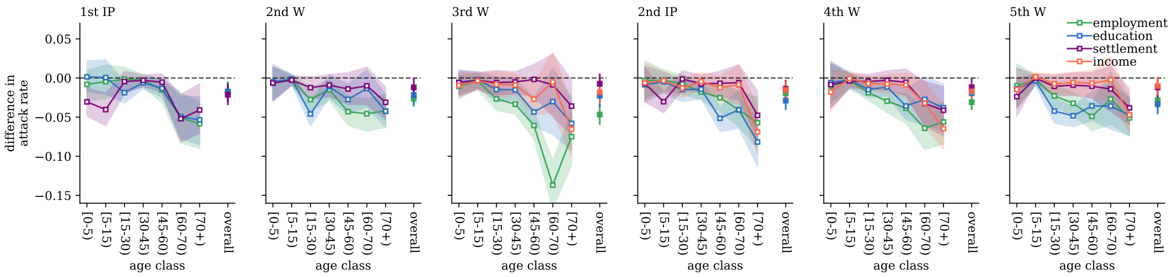

Supplementary Figure 20: Difference in the attack rate by age as predicted by the *classical* ($Mw/C_{i,j}$) and the *extended* ($Mw/C_{di,j}$) model, when different the dimensions are considered. Results are shown for each of the periods (*columns*). Epidemiological parameters: $\mu = 0.4, \epsilon = 0.25$, and $R_0 = 2.5$. Simulations start with $I_0 = 5$ initial infectious seed. Results were computed over 1000 simulations.

.

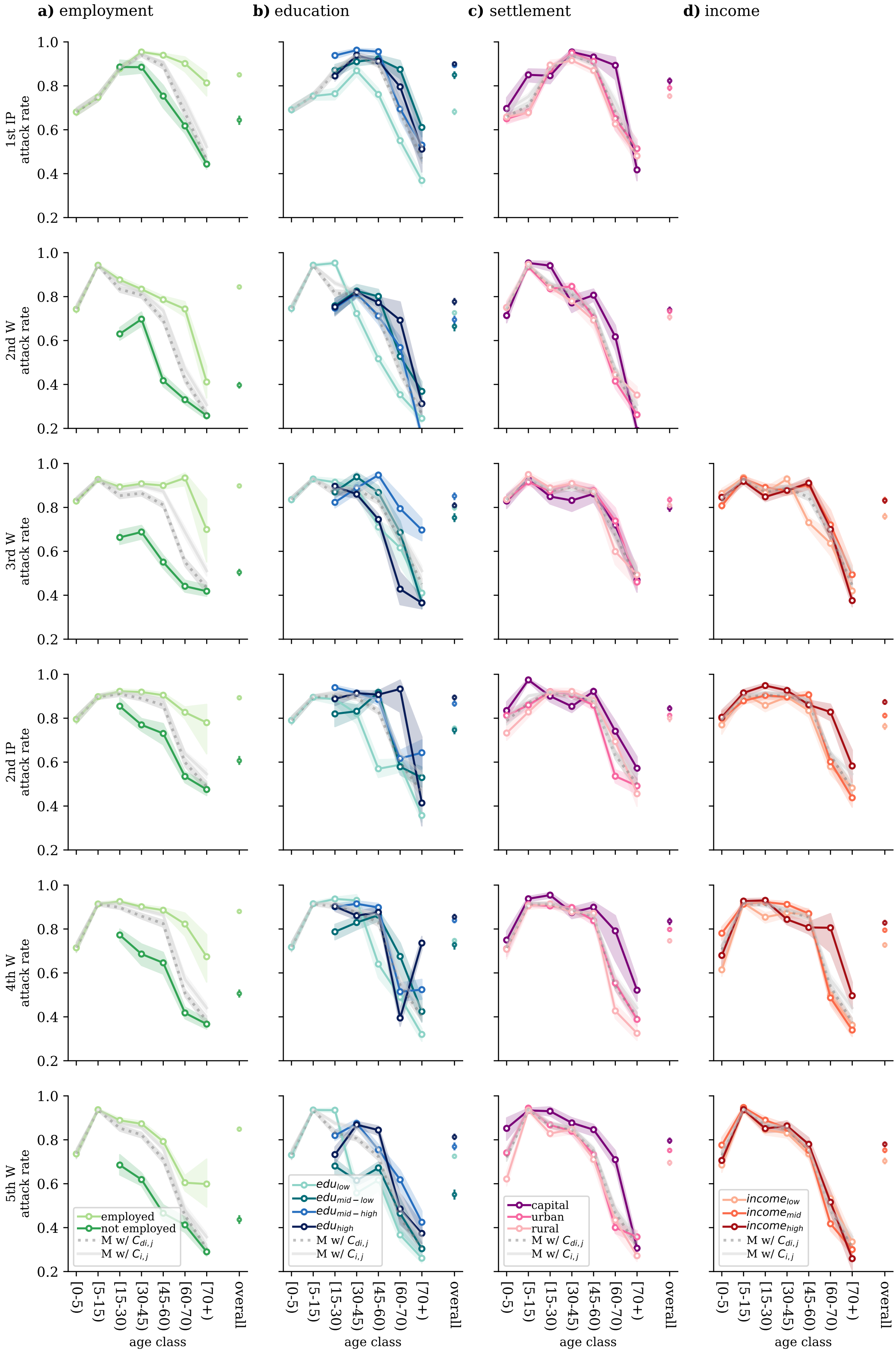

Supplementary Figure 21: Attack rate by age and employment situation, education level, settlement and income (*columns*), in different periods (*rows*). Epidemiological parameters: $\mu = 0.4, \epsilon = 0.25$, and $R_0 = 2.5$. Simulations start with $I_0 = 5$ initial infectious seed. Results were computed over 1000 simulations.

7.1.1 Sensitivity analysis of R_0

Here we conduct a sensitivity analysis by reiterating the analysis with varying values of the basic reproductive number (R_0). Figure 22 illustrates the variation in the difference of attack rates, across *age groups* and *overall* (*rows*) as predicted by the *classical* ($Mw/C_{i,j}$) and *extended* ($Mw/C_{di,j}$) models, as R0 increases. These findings indicate that, as the epidemic intensifies with higher R0 values, the difference in attack rates exhibits a decreasing trend with lower uncertainty for younger age groups. Conversely, most older age groups display an increasing trend, also with lower uncertainty. The overall difference in the attack rate of the population shows an increasing trend with lower uncertainty. This sensitivity analysis underscores significant discrepancies between the two models in numerous scenarios, highlighting that the *classical SEIR* model tends to overestimate the attack rate, particularly when compared to the *extended SEIR* model, which incorporates employment.

Furthermore, Figures 23 and 24 present the attack rates by age for different subgroups of the population respectively for $R_0 = 1.5$ and $R_0 = 3.5$. Here the results show that the increased transmissibility of the epidemic impacts equally each group of the population by leaving almost unchanged the differences among the subgroups of the population.

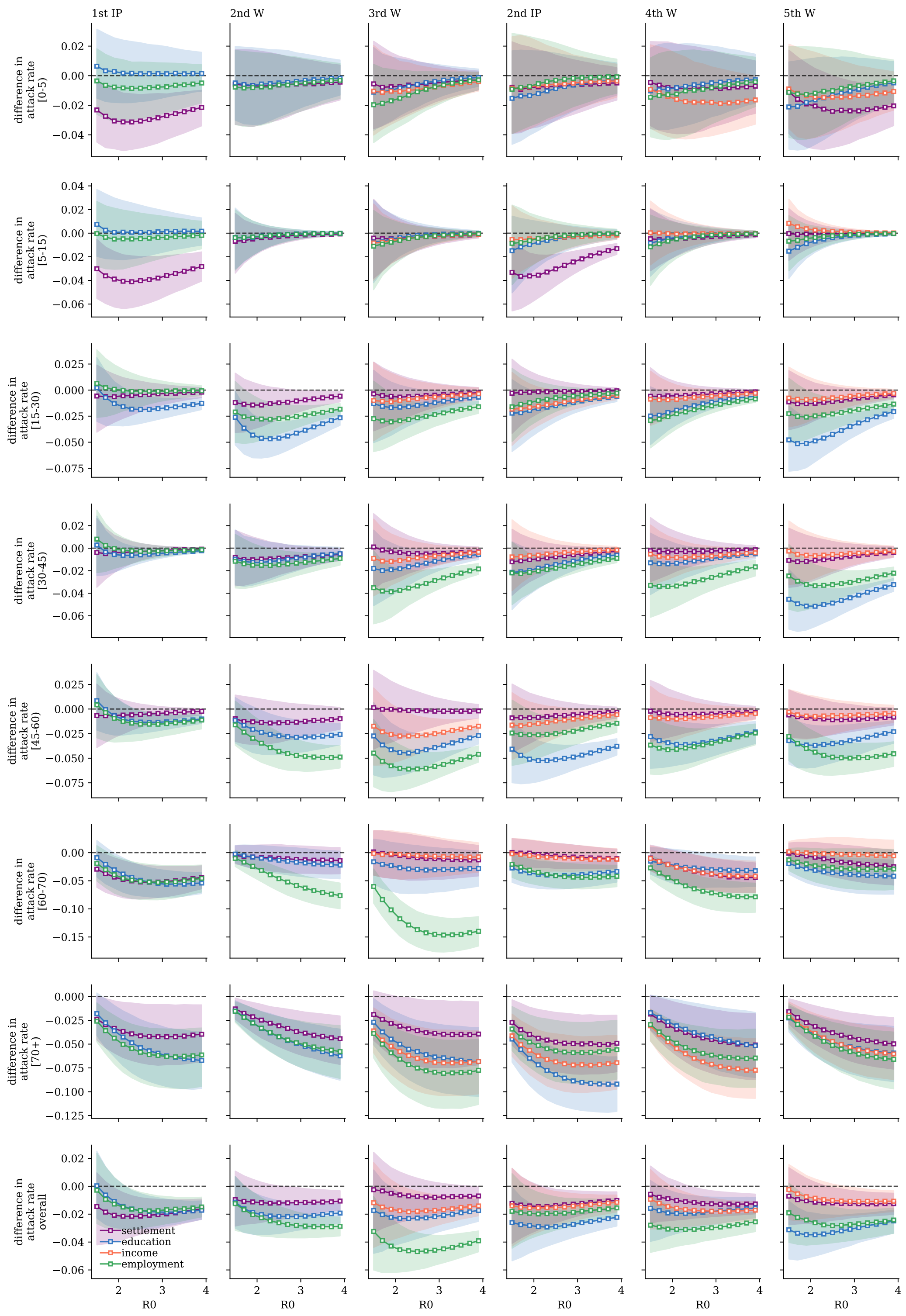

Supplementary Figure 22: Difference in the attack rate by age and overall (*rows*) as predicted by the *classical* ($Mw/C_{i,j}$) and the *extended* ($Mw/C_{di,j}$) model, for different value of R_0. Results are shown for each of the periods (*columns*). Epidemiological parameters: $\mu = 0.4, \epsilon = 0.25$. Simulations start with $I_0 = 5$ initial infectious seed. Results were computed over 1000 simulation.

.

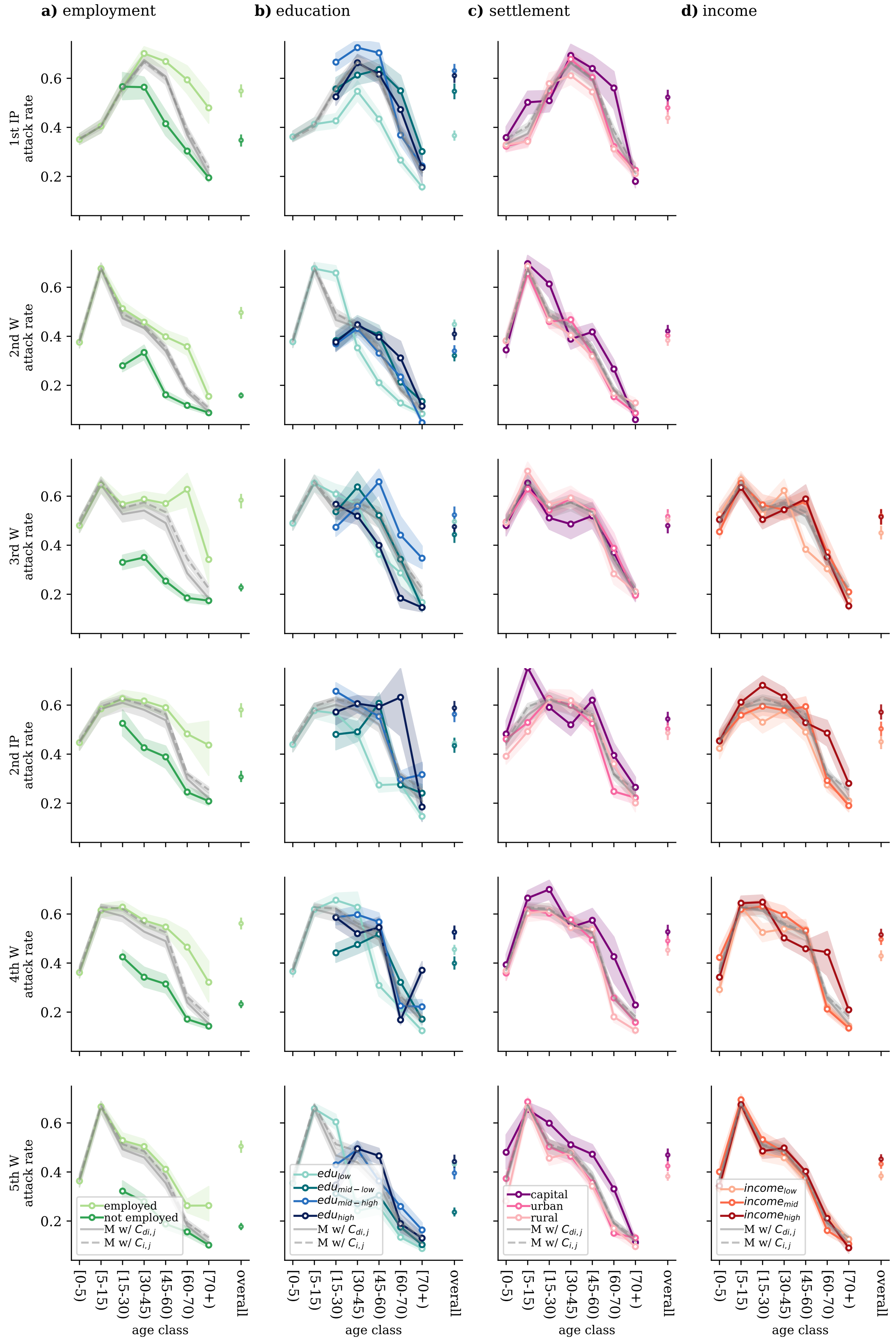

Supplementary Figure 23: Attack rate by age and employment situation, education level, settlement and income (*columns*), in different periods (*rows*). Epidemiological parameters: $\mu = 0.4, \epsilon = 0.25$, and $R_0 = 1.5$. Simulations start with $I_0 = 5$ initial infectious seed. Results were computed over 1000 simulations.

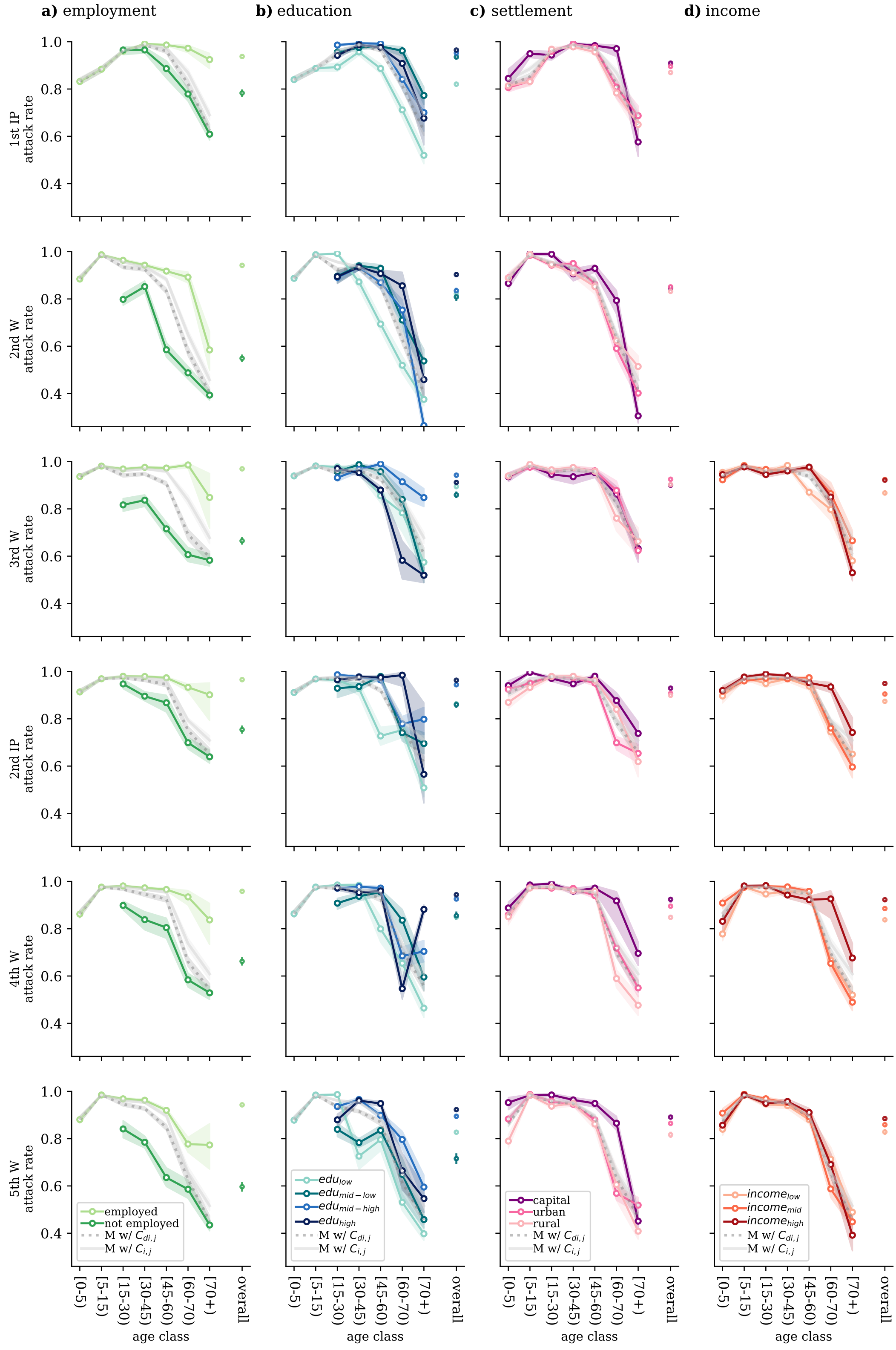

Supplementary Figure 24: Attack rate by age and employment situation, education level, settlement and income (*columns*), in different periods (*rows*). Epidemiological parameters: $\mu = 0.4, \epsilon = 0.25$, and $R_0 = 3.5$. Simulations start with $I_0 = 5$ initial infectious seed. Results were computed over 1000 simulations.

7.2 Impact of different vaccination uptake

In order to show the impact of different vaccination uptake here we show the *averted attack rate* by age due to vaccination (Fig. 25). Specifically, we show the difference among the attack rate by age, for the different subgroups as predicted by the *extended SEIR* in the non-vaccination scenario with respect to the one in which individuals get vaccinated according to their age and subgroup- as shown in Fig. 19.

The findings from the additional periods support the observations discussed in the main text. Indeed, Fig. 25 clearly shows that vaccination benefits are disproportionately advantageous for more privileged population groups.

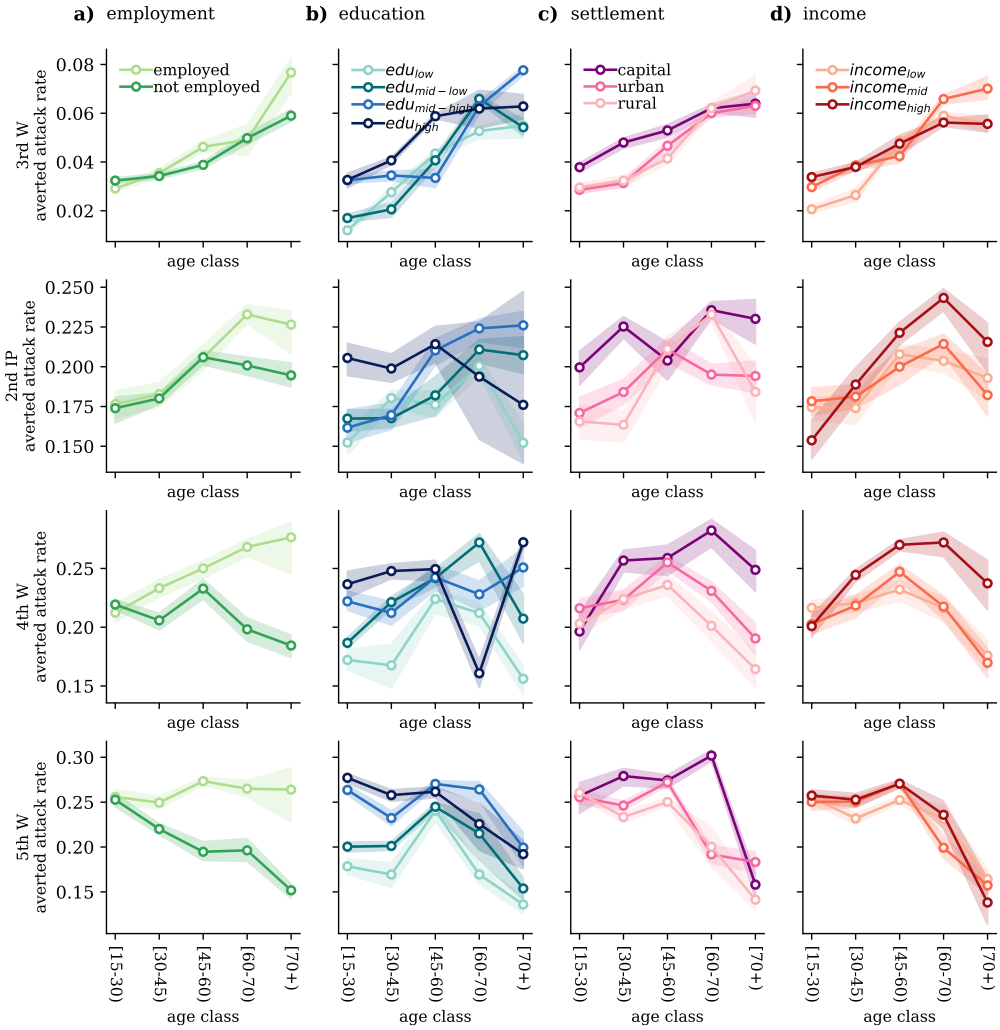

Supplementary Figure 25: Difference in attack rate due to vaccination by age and employment situation, education level, settlement and income (*columns*), in different periods (*rows*). Epidemiological parameters: $\mu = 0.4, \epsilon = 0.25$, and $R_0 = 2.5$. Efficacy of vaccination against infection (g_1) is set to 0.6. Simulations start with $I_0 = 5$ initial infectious seed. Results were computed over 1000 simulations.

7.2.1 Sensitivity analysis of vaccination efficacy against infection (g_1)

To investigate the robustness of the results presented above we conduct a sensitivity analysis by varying the efficacy of vaccination (g_1). Namely, Figure 26 presents results for simulations with g_1 set to 0.2, while Figure 27 displays outcomes for simulations with g_1 set to 0.9.

By design, $g_1 = 0$ corresponds to the non-vaccination scenario, thus the disparity in attack rate differences attributable to vaccination diminishes towards zero as g_1 decreases. Consequently, as expected, when g_1 is set to 0.2 (Fig. 26), the overall magnitude of the difference becomes smaller. Nevertheless, discernible and meaningful differences persist among subgroups of the population. Conversely, when g_1 is set to 0.9 (Fig. 27), both the overall magnitude of the difference and the disparities among subgroups increase, accentuating the distinctions among population groups that are undergoing higher vaccination rates.

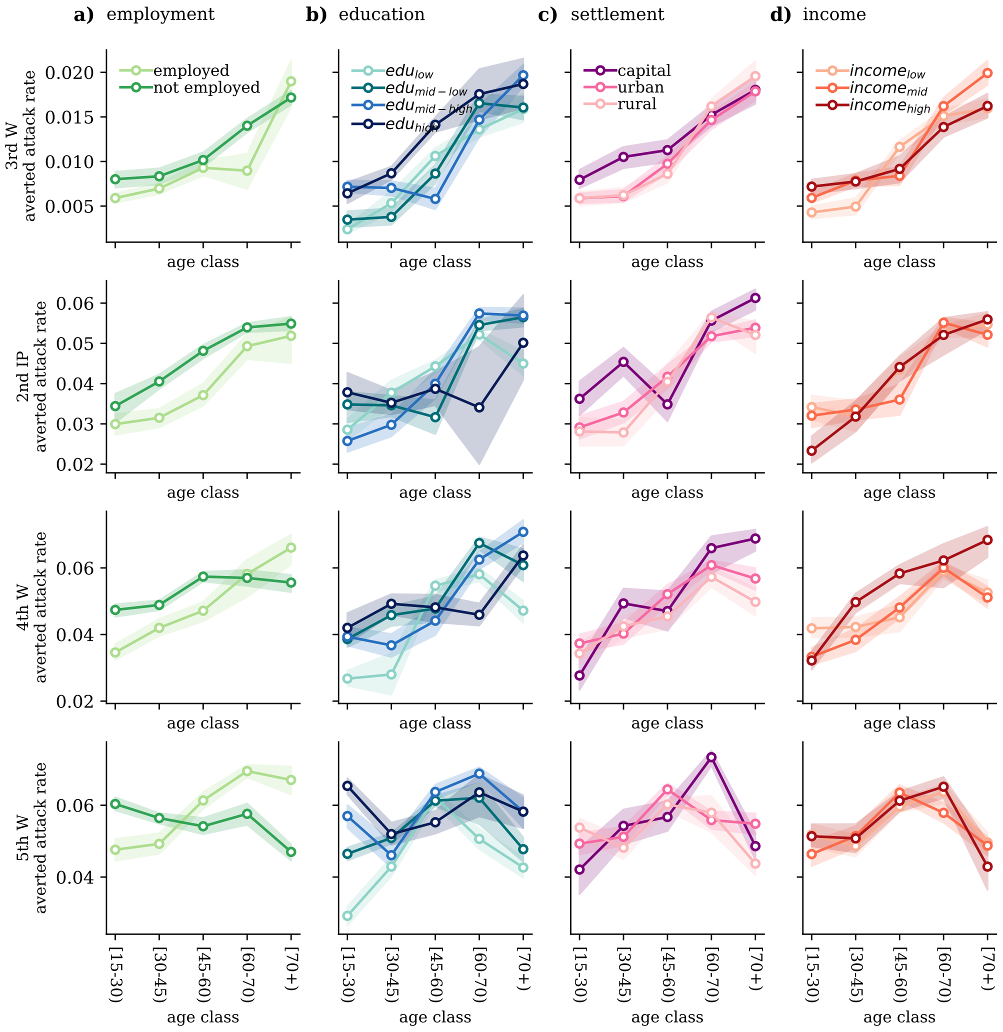

Supplementary Figure 26: Difference in attack rate due to vaccination by age and employment situation, education level, settlement and income (*columns*), in different periods (*rows*). Epidemiological parameters: $\mu = 0.4, \epsilon = 0.25$, and $R_0 = 2.5$. Efficacy of vaccination against infection (g_1) is set to 0.2. Simulations start with $I_0 = 5$ initial infectious seed. Results were computed over 1000 simulations.

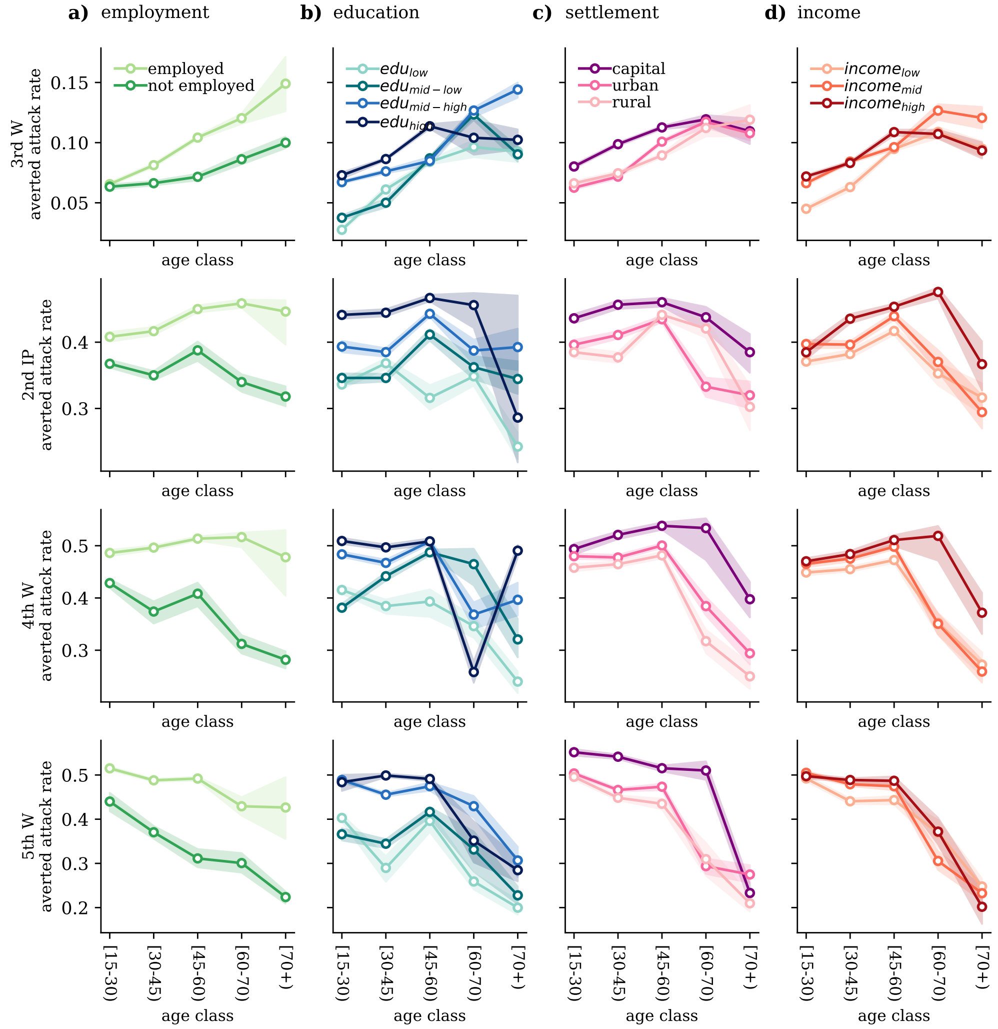

Supplementary Figure 27: Difference in attack rate due to vaccination by age and employment situation, education level, settlement and income (*columns*), in different periods (*rows*). Epidemiological parameters: $\mu = 0.4, \epsilon = 0.25$, and $R_0 = 2.5$. Efficacy of vaccination against infection (g_1) is set to 0.9. Simulations start with $I_0 = 5$ initial infectious seed. Results were computed over 1000 simulations.

8 Model Calibration

We calibrate a SEIRD model with vaccination by modelling differences in contact patterns and vaccination uptake among employed and not-employed individuals belonging to different education levels. In particular, we calibrate the free parameters of the model using an Approximate Bayesian Computation (ABC) technique [9, 14]. First, we define the prior distributions of the free parameters $P(\theta)$, a number of accepted sets N, an error metric $m(E, E')$, and a tolerance δ. We start sampling a set of parameters θ from $P(\theta)$, and generate an instance of the model using these parameters. Then, using the chosen error metric we compare an output quantity E' of the model with the corresponding real quantity E: if $m(E, E') < \delta$ then we accept the set θ, otherwise we reject it. We repeat this accept/reject step until N parameter sets are accepted. The empirical distribution of the accepted sets is an approximation of their real posterior distribution. Finally, we generate an ensemble of possible epidemic trajectories sampling parameter sets from the posterior distributions. In this work, we consider the following free parameters and prior distributions:

- the transmission rate parameter β: the prior distribution is set to $U(0.02, 0.15)$
- the delay in reporting deaths Δ: the prior distribution is set to $U(5, 20)$
- the initial recovered population R: the prior distribution is set to $U(700K, 3500K)$
- the initial exposed population E: the prior distribution is set to $U(100, 3K)$
- the initial infected population I: the prior distribution is set to $U(200, 9K)$

Given the total number of individuals Exposed, Infected and Recovered at $t = 0$, these are assigned to age group, employment situation and income level according to the population distribution.

We calibrate our model on the aggregate number of daily deaths from 09/2021 to 01/2022. For simplicity, as the percentage of those who were vaccinated was quite stable [13] in the period considered we assume that the population got vaccinated at time 0. As an error metric, we use the *Median Absolute Percentage Error (MdAPE)*. We also set the number of accepted sets $N = 3000$ and the tolerance $\delta = 0.35$ In Fig. 28 are shown the posterior distributions of the parameters calibrated through the ABC rejection algorithm.

The fixed parameters of the model have been informed from the literature. In particular:

- the efficacy of the vaccine against infection and against death, is modelled as a normal distribution with mean respectively $g_1 = 0.7$, $g_2 = 0.8$, and standard deviation 0.05 [15, 12]. This choice has been made to account for the variability of the efficacy of the different vaccination types and against the different variants.
- the recovery rate $\mu = 1/2.5$ [4, 2, 8]
- the incubation period $\epsilon = 1/4$ [4, 2, 8]
- the infection fatality rate by age IFR_i is set as in Table 9 [11]

In Fig. 28 we report the number of daily real and simulated deaths (median and IQR).

Age group	IFR
[0-5)	0.001%
[5-15)	0.001%
[15-30)	0.005%
[30-45)	0.02%
[45-60)	0.2%
[60-70)	0.7%
[70+)	3.1%

Supplementary Table 9: Infection Fatality Rate by age group

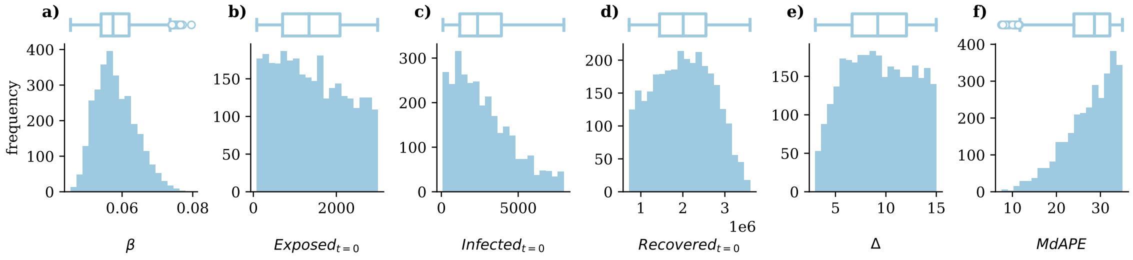

Supplementary Figure 28: **(a-e)**Posterior distribution of the calibrated parameters of the model **(f)** Distribution of the MdAPE (ϵ).

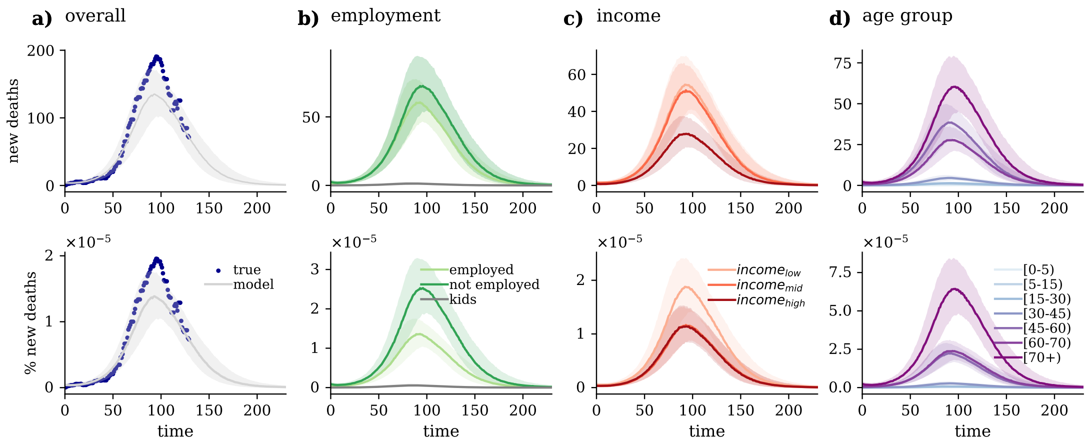

Supplementary Figure 29: **(a-d)** Number of daily deaths real (blue dots) and simulated (grey) overall (a), by education (b), employment (c), and age group (d).**(e-h)** Percentage of daily deaths real(red line) and simulated (grey) overall (e), by education (f), employment (g), and age group(h). Results refer to the median and IQR of 1000 simulations.

References

[1] Sergio Arregui et al. "Projecting social contact matrices to different demographic structures". In: *PLOS Computational Biology* 14.12 (Dec. 2018), pp. 1–18. DOI: 10.1371/journal.pcbi.1006638. URL: https://doi.org/10.1371/journal.pcbi.1006638.

[2] Jantien A Backer, Don Klinkenberg, and Jacco Wallinga. "Incubation period of 2019 novel coronavirus (2019-nCoV) infections among travellers from Wuhan, China, 20–28 January 2020". In: *Eurosurveillance* 25.5 (2020), p. 2000062.

[3] Julie C Blackwood and Lauren M Childs. "An introduction to compartmental modeling for the budding infectious disease modeler". In: (2018).

[4] Nicolò Gozzi et al. "Anatomy of the first six months of COVID-19 Vaccination Campaign in Italy". In: *PLoS Computational Biology* 18.5 (2022), e1010146.

[5] Eleonora Dal Grande et al. "Health estimates using survey raked-weighting techniques in an Australian population health surveillance system". In: *American journal of epidemiology* 182.6 (2015), pp. 544–556.

[6] Mackenzie Hamilton, Jesse Knight, and Sharmistha Mishra. "Examining the Influence of Imbalanced Social Contact Matrices in Epidemic Models". In: *American Journal of Epidemiology* (Sept. 2023), kwad185. ISSN: 0002-9262. DOI: 10.1093/aje/kwad185. eprint: https://academic.oup.com/aje/advance-article-pdf/doi/10.1093/aje/kwad185/51608759/kwad185.pdf. URL: https://doi.org/10.1093/aje/kwad185.

[7] Matt J. Keeling and Pejman Rohani. *Modeling Infectious Diseases in Humans and Animals*. Princeton: Princeton University Press, 2008. Chap. 3. ISBN: 9780691116174. URL: http://www.jstor.org/stable/j.ctvcm4gk0.

[8] Stephen M Kissler et al. "Projecting the transmission dynamics of SARS-CoV-2 through the postpandemic period". In: *Science* 368.6493 (2020), pp. 860–868.

[9] Amanda Minter and Renata Retkute. "Approximate Bayesian Computation for infectious disease modelling". In: *Epidemics* 29 (2019), p. 100368.

[10] Max Roser. "What is the COVID-19 Stringency Index?" In: *Our World in Data* (2021). https://ourworldindata.org/[illegible] explained-covid19-stringency-index.

[11] Henrik Salje et al. "Estimating the burden of SARS-CoV-2 in France". In: *Science* 369.6500 (2020), pp. 208–211.

[12] Julia Shapiro et al. "Efficacy estimates for various COVID-19 vaccines: what we know from the literature and reports". In: *MedRxiv* (2021), pp. 2021–05.

[13] Statista. *Hungary: Number of people vaccinated against COVID-19*. https://www.statista.com/statistics/1196109/hungary-number-of-people-vaccinated-against-covid-19/. Accessed: May 9, 2023. 2023.

[14] Mikael Sunnåker et al. "Approximate bayesian computation". In: *PLoS computational biology* 9.1 (2013), e1002803.

[15] Zoltán Vokó et al. "Nationwide effectiveness of five SARS-CoV-2 vaccines in Hungary—the HUN-VE study". In: *Clinical Microbiology and Infection* 28.3 (2022), pp. 398–404.

[16] Jacco Wallinga, Peter Teunis, and Mirjam Kretzschmar. "Using Data on Social Contacts to Estimate Age-specific Transmission Parameters for Respiratory-spread Infectious Agents". In: *American Journal of Epidemiology* 164.10 (Sept. 2006), pp. 936–944. ISSN: 0002-9262. DOI: 10.1093/aje/kwj317. eprint: https://academic.oup.com/aje/article-pdf/164/10/936/385238/kwj317.pdf. URL: https://doi.org/10.1093/aje/kwj317.

[17] James Wambua et al. "The influence of COVID-19 risk perception and vaccination status on the number of social contacts across Europe: insights from the CoMix study". In: *BMC Public Health* 23.1 (2023), p. 1350.
